# Drug transporters OAT1 and OAT3 have specific effects on multiple organs and gut microbiome as revealed by contextualized metabolic network reconstructions

**DOI:** 10.1038/s41598-022-21091-w

**Published:** 2022-10-31

**Authors:** Neema Jamshidi, Sanjay K. Nigam

**Affiliations:** 1 Department of Radiological Sciences, University of California, Los Angeles, Los Angeles, CA USA; 2 Institute of Engineering in Medicine, University of California, San Diego, La Jolla, CA USA; 3 Departments of Pediatrics and Medicine (Nephrology), University of California, San Diego, La Jolla, CA USA

**Keywords:** Biochemistry, Computational biology and bioinformatics, Kidney, Metabolism, Systems biology, Biochemical networks, Computer modelling, Genetic interaction, Systems analysis

## Abstract

In vitro and in vivo studies have established the organic anion transporters OAT1 (SLC22A6, NKT) and OAT3 (SLC22A8) among the main multi-specific “drug” transporters. They also transport numerous endogenous metabolites, raising the possibility of drug-metabolite interactions (DMI). To help understand the role of these drug transporters on metabolism across scales ranging from organ systems to organelles, a formal multi-scale analysis was performed. Metabolic network reconstructions of the omics-alterations resulting from *Oat1* and *Oat3* gene knockouts revealed links between the microbiome and human metabolism including reactions involving small organic molecules such as dihydroxyacetone, alanine, xanthine, and *p*-cresol—key metabolites in independent pathways. Interestingly, pairwise organ-organ interactions were also disrupted in the two *Oat* knockouts, with altered liver, intestine, microbiome, and skin-related metabolism. Compared to older models focused on the “one transporter-one organ” concept, these more sophisticated reconstructions, combined with integration of a multi-microbial model and more comprehensive metabolomics data for the two transporters, provide a considerably more complex picture of how renal “drug” transporters regulate metabolism across the organelle (e.g. endoplasmic reticulum, Golgi, peroxisome), cellular, organ, inter-organ, and inter-organismal scales. The results suggest that drugs interacting with OAT1 and OAT3 can have far reaching consequences on metabolism in organs (e.g. skin) beyond the kidney. Consistent with the Remote Sensing and Signaling Theory (RSST), the analysis demonstrates how transporter-dependent metabolic signals mediate organ crosstalk (e.g., gut-liver-kidney) and inter-organismal communication (e.g., gut microbiome-host).

## Introduction

A fundamental challenge in medicine is the interpretation of systemic responses to perturbations of individual genes or sets of genes (or gene products). In particular, so-called drug transporters are understudied from a systems biology perspective. Along with mono-specific and oligo-specific transporters, these multi-specific SLC and ABC drug transporters are now known to have important roles in endogenous metabolism. Thus, transporters are increasingly considered to be therapeutic targets for treatment of metabolic diseases. For example, the targeting by gliflozins of the sodium-glucose transporter SGLT2 (SLC5A2) is becoming a widely utilized therapy for Type 2 diabetes^1^.

Multi-specific transporters (e.g. Organic anion transporter 1, OAT1 or SLC22A6, originally identified as NKT; Organic anion transporter 3, OAT3 or SLC22A8; Breast cancer resistance protein, BCRP or ABCG2) handle a remarkably wide range of drugs^2–4^. The Food and Drug Administration (FDA) has emphasized the clinical importance of understanding whether newly approved drugs are transported by these and a few other multi-specific SLC and ABC transporters^5, 6^. Although they have immense pharmacokinetic importance, there is a growing appreciation of their endogenous roles in the transport of key metabolites and signaling molecules^5^. Many of the metabolites handled by “drug” transporters originate in the gut microbiome in physiological and pathophysiological circumstances^7^. Therefore, understanding the local and systemic metabolic consequences of disruption of multi-specific drug transporters (as well as oligo-specific and mono-specific SLC and ABC transporters)—including metabolism dependent upon small organic molecules originating in the gut microbes—is of tremendous practical import.

OAT1 and OAT3 are particularly attractive for integrating metabolism across multiple drug transporters, multiple organs, the gut microbiome, and multiple scales^3, 4^. From the organ and inter-organ perspective, there is now considerable evidence regarding the key roles of OATs in kidney proximal tubule regulation of plasma levels as well as renal and urine levels of fatty acids, uric acid, vitamins, bile acids, and prostaglandins, as well wide range of other metabolites and signaling molecules^8^. Intracellularly, OATs help regulate cytosolic levels of “classic” Krebs Cycle metabolites such as alpha-ketoglutarate^9^. On an inter-organismal scale, OATs appear to be one of the major systemic routes for elimination of numerous gut microbiome-derived metabolites; once inside the cells of the kidney, some of these metabolites also activate nuclear receptor signaling, leading to the expression of other transporters and enzymes^10–12^. Given this coming together of recent in vitro and in vivo experimental evidence from several groups, suggesting the impact of OAT functioning upon multiple biological scales (including intracellular metabolism, organ metabolism, inter-organ metabolism, and inter-organismal metabolism), *Oat1* and *Oat3* seem an ideal set of genes for condition-specific genome-scale metabolic reconstruction at multiple scales.

**Figure 1:**
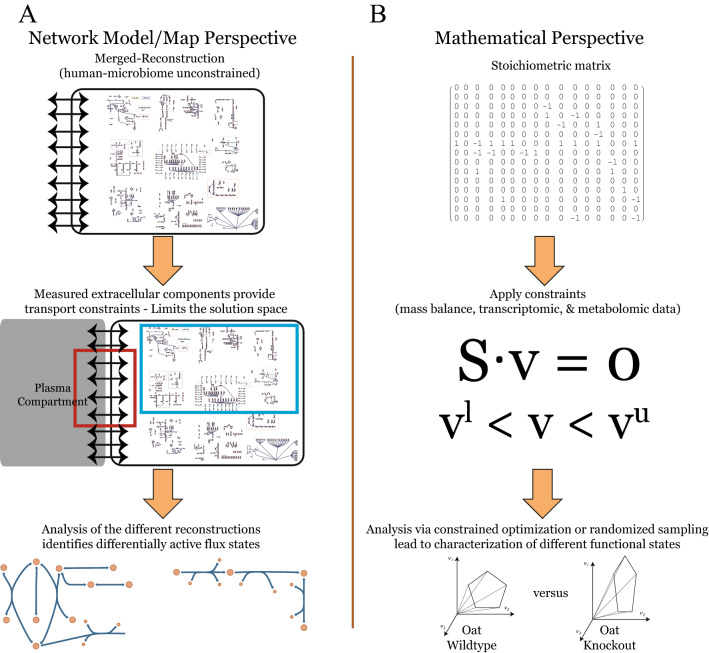
Data integration and analysis workflow. Panels (**A**) and (**B**) show two perspectives of the same process: the former outlines the graphical depiction of the integration and modeling approach; the latter provides a high-level outline of the key modeling implementation and analysis steps. These are: (1) The conversion process from a reconstruction to a computable model involves translating a map of biochemical reactions into the stoichiometric matrix. (2) This stoichiometric matrix then used to perform constraint-based optimization using experimental data (e.g. omics data from WT versus KO). 3) Finally, simulations are performed in order to define the in silico phenotypes, which are characterized in the accompanying figures and tables. More specifically, in this study the global human-microbe network reconstruction is constrained with condition-specific data for WT and (OAT1 and OAT3) KO mice; these are then compared in order to identify the systemic metabolic differences resulting from loss of transport function for OAT1 and OAT3. These steps are described in the “Methods”. Please also see Fig. 2 and Supplemental Fig. S1. *S* stoichiometric matrix, *v* reaction flux vector, *c* objective vector, *v*^*u*^ reaction upper bound vector, *v*^*l*^ reaction lower bound vector.

Genome-scale metabolic network reconstructions provide a means to carry out multi-omic, data-driven model construction, that enable network flux estimations to provide an assessment of the metabolic capabilities of the cells (Fig. 1). Most constraint-based reconstruction analysis (COBRA) methods do not require any parameterization; thus there does not need to be any ‘training’ on the data, and the calculated results are not subject to overfitting^13, 14^. Gene-protein-reaction relationships provide the means to map transcriptomic data onto the metabolic network. Data integration algorithms provide the means to generate context-specific models using transcriptomic data (as well as any available proteomic or metabolomic data)^15, 16^. Beginning over a decade ago, physiologically-relevant results were obtained with this general approach using earlier versions of human genome annotation and incomplete representation of transporters to integrate transcriptomics and very limited metabolomics data in a "one transporter-one organ" approach. Recent approaches have enabled the generation of useful condition-specific models capable of simulating the effects of drug-induced, as well as genetic, perturbations with experimental validation^17–21^. The models in turn can be used to test current notions and generate new, testable hypotheses^22–25^.

Due to limitations in scope of the metabolic reconstructions as well as relatively limited throughput of metabolomics, many complex inter-organ and gut-microbiome interactions were not detectable a decade ago. However, through the use of much more expansive plasma metabolomic profiling of *Oat1* and *Oat3* knockouts (KO) in conjunction with the current iteration of the human metabolic network reconstruction—and with the incorporation of multiple microbial models—we are now able expand the scope of the systemic alterations resulting from the loss of function of these transporters. For the first time, the reconstruction explores inter-organ interactions as well as the role of the gut microbiome in OAT-regulated metabolism. The results, while consistent with previous "one transporter-one organ" analysis, reveal many novel interactions across multiple organs and multiple scales. Furthermore, because of the intimate connection between gut microbe-derived metabolites and renal OAT1 and OAT3 function in the setting of kidney disease^3^, this strategy could set the stage for a much more formal and transparent understanding of the systems biology of chronic kidney disease (CKD).

Here we use a multi-omics-based systems biology approach to interpret organ-specific as well as systemic responses (including gut microbe responses) to the organic anion transporters, OAT1 and OAT3 in KO mice. Our strategy reconciles intra-cellular (including inter-organelle) metabolism with multi-organ (e.g. kidney-liver) metabolism as well as inter-organismal (e.g. host-gut microbe) metabolism using a human metabolic network reconstruction, Recon3D^26^, in conjunction with a representative microbial model (composed of an amalgam of multiple microbial models including, *Bacillus subtilis, Lactococcus lactis,* and multiple strains of *Escherichia coli*) to construct multiple host organ-microbiome models. The resulting OAT1 and OAT3 wildtype (WT) and KO models are then used to evaluate aspects of function within the whole organism in relation to: (1) kidney-specific functions; (2) multi-organ and microbiome interactions; (3) intracellular reaction fluxes and organelle-associated functions; and (4) metabolic pathways (canonical and context-specific). The results indicate how the changes in function of a single transporter, as well as multiple transporters, manifest in specific organelle, cellular, organ, inter-organ, systemic, and inter-organismal (host-gut microbe) alterations in metabolism. While the centrality of the transport function OAT1 and OAT3 in the gut (including microbiome)-liver-kidney axis is reaffirmed, previously unrecognized connections to reactions in other tissues, such as those predominantly occurring in skin, are revealed. The results support the Remote Sensing and Signaling Theory (RSST) of inter-organ and inter-organismal communication via small organic molecules that interact with "drug" transporters, "drug" metabolizing enzymes and nuclear receptors^3, 5, 10, 12^.

## Results

**Figure 2:**
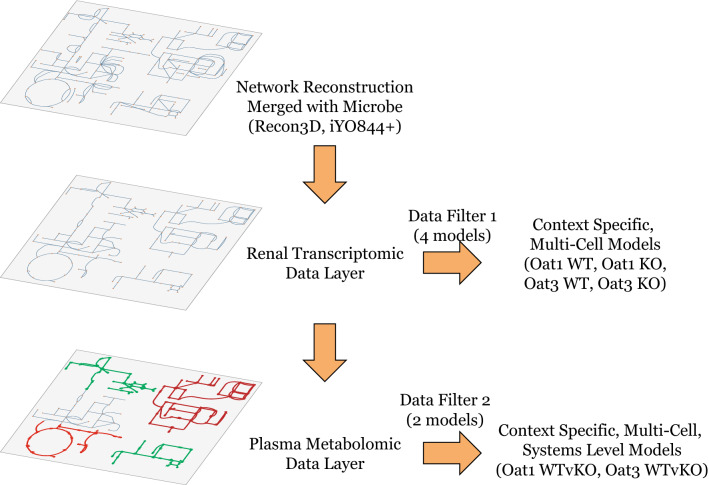
Schematic of study design. Shown here is an outline of the workflow to generate the context-specific models for the OAT1 and OAT3 “transcriptome model” and “transcriptome plus metabolome” models. As described in Methods, a human metabolic reconstruction (Recon3D) was merged with a representative microbiome metabolic reconstruction. The first tier (‘Data Filter 1’) involves generation of context-specific models for OAT1 and OAT3 WT and KO mice using renal transcriptomic data. The second tier (‘Data Filter 2’) uses targeted plasma metabolomic data to generate sets of WT versus KO comparison models. Since the plasma metabolome includes inter-organ interactions (uptake, elimination, and metabolism) across all organs in the body, alterations (synthesis and degradation) due to non-renal metabolism can be inferred using independently defined metabolic objectives (see “Methods” and main text).

**Figure 3:**
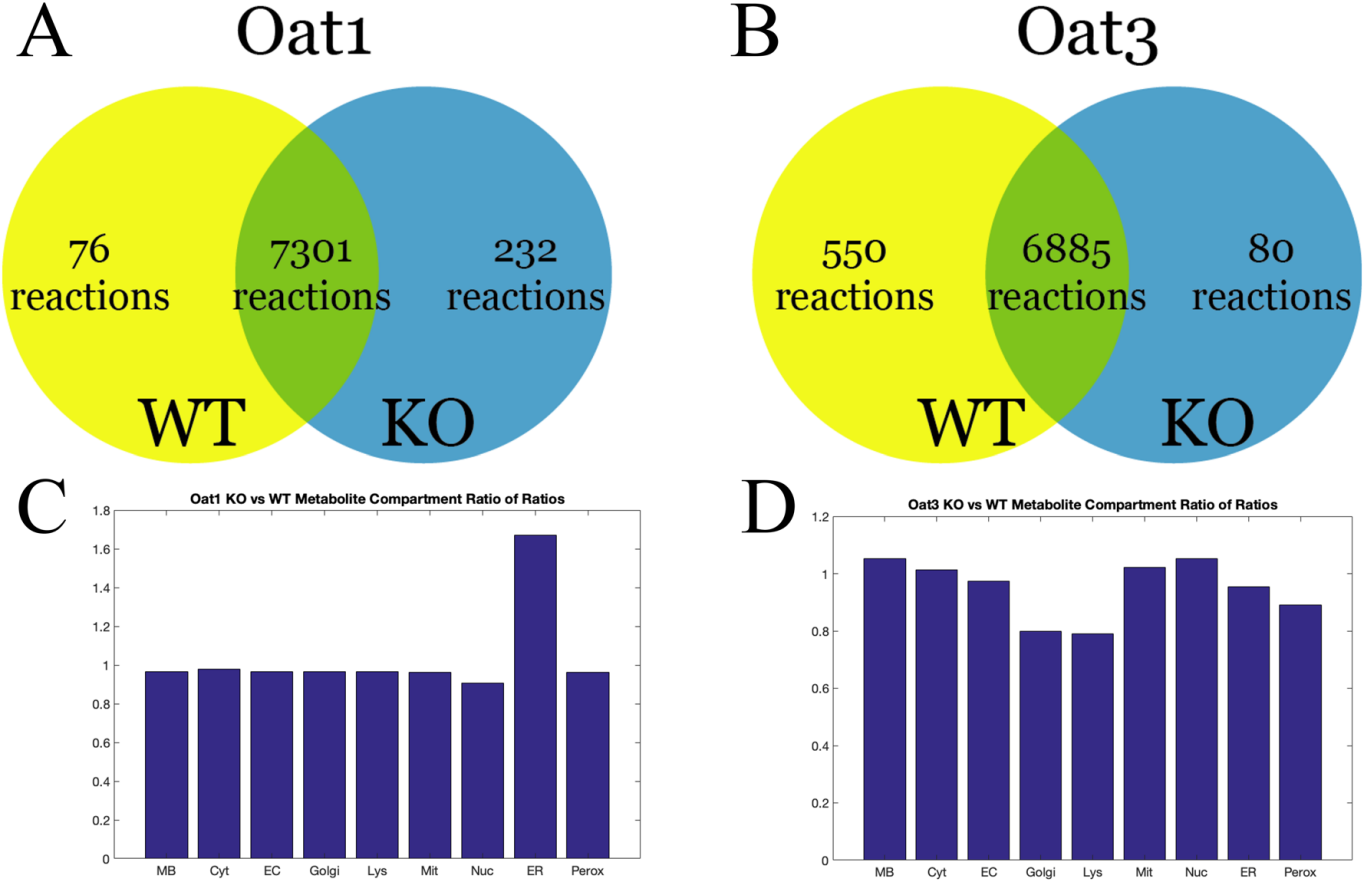
Comparison of metabolic reactions and organelle metabolite content between WT and OAT1 and OAT3 KO mice. Comparison between the renal transcriptome-constrained models revealed overlap between the WT and KO, as expected, but it is important to note there exist notable differences. In particular, there are metabolic reactions that are “unique” or specific to the WT but not to one or both knockouts, and vice-versa. These differences are manifest in part by the organelle-specific metabolite composition changes (e.g. endoplasmic reticulum) in the KO mice reconstructions. The organelle-based differences reflecting different pathways of metabolism are noted in OAT1 versus OAT3. (**A**) OAT1 WT and KO Venn diagram summary of the kidney/microbiome model. The relatively larger number of unique KO model reactions suggests that in order to compensate for the loss of OAT1, the kidney cells must rely on a larger set of reactions associated with certain organelles. (**B**) OAT3 WT and KO Venn diagram summary of the kidney/microbiome model. (**C**) Ratio of the relative number of unique metabolites in OAT1 KO versus WT models across all 9 compartments. There is a significant increase in the number of metabolites in the endoplasmic reticulum (Chi-square p < 0.001) of the knockouts; these largely reflect the 232 unique knockout model reactions. (**D**) Ratio of the relative number of unique metabolites in OAT3 KO versus WT models across all 9 compartments. In contrast to the OAT1 WT versus KO, the OAT3 KO has a significantly lower number of metabolites (Chi-square p < 0.01) in several compartments, namely the Golgi apparatus, lysosome, and peroxisome; these changes are also consistent with the relatively larger number of unique reactions in the WT model (**B**). *MB* microbiome, *Cyt* cytosol, *EC* extracellular, *Gol* Golgi, *Lys* lysosome, *Mit* mitochondria, *Nuc* nucleus, *ER* endoplasmic reticulum, *Perox* peroxisome.

To briefly summarize the reconstruction strategy that follows, renal transcriptomic and untargeted plasma metabolomic data from *Oat1* and *Oat3* WT and KO mice were analyzed using an integrated human-microbiome genome-scale metabolic network model, starting with Recon3D merged with a representative multi-microbial model (Fig. 2). A two-tier model construction approach was employed. The first tier consisted of creating context-specific models of the kidney-gut microbiome using renal transcriptomics data (Fig. 3), and the second tier involved incorporating plasma metabolomics data. The analysis can be viewed as a ‘Data Filter’ (Figs. 2), resulting in four context-specific metabolic network models (two WT/KO pairs). Comparisons of overall content and the relative composition of the networks enables an assessment of: (1) the similarities and differences in the WT versus KO models based on reaction composition, including cross-comparison between *Oat1* and *Oat3*, (2) functional comparison between WT and KO for *Oat1* and *Oat3*, respectively, based on context specific network reconstruction content and steady state flux comparisons.

### Transcriptome analysis

(Fig. 2, Data Filter 1): Context-specific models of the kidney-gut microbiome connections using transcriptomic data from the *Oat1* and *Oat3* WT and KO mice.

In spite of overlap in loss of function resulting from knockout of each of the transporters—for example, uric acid and diuretics are taken up into the kidney proximal tubule cells by both transporters^27, 28^—it has recently been clearly established, in contrast to earlier views, that there are many independent metabolic functions for OAT1 and OAT3^29, 30^.

**Figure 4:**
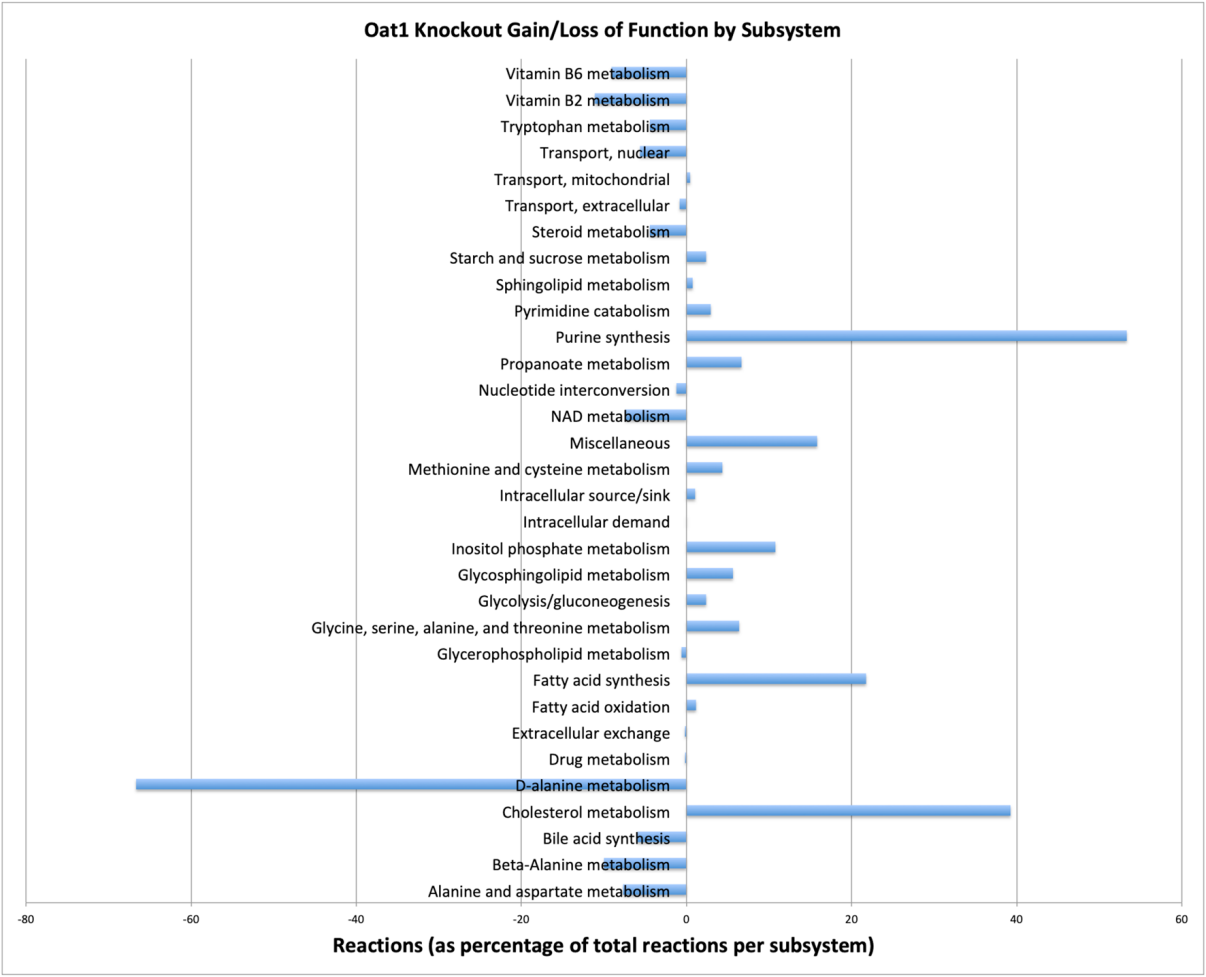
Changes in mutually exclusive reactions in the WT versus OAT1 KO models according to metabolic sub-systems shown as a percentage of the total number of reactions in each Recon3D sub-system. The changes in sub-systems are not uni-directional; some sub-systems increase and others decrease. Notably, in the Oat1KO model there is an increase in purine metabolism, cholesterol metabolism, and fatty acid synthesis, while alanine and aspartate metabolism and Vitamin B metabolism sub-systems are noted to decrease.

**Figure 5:**
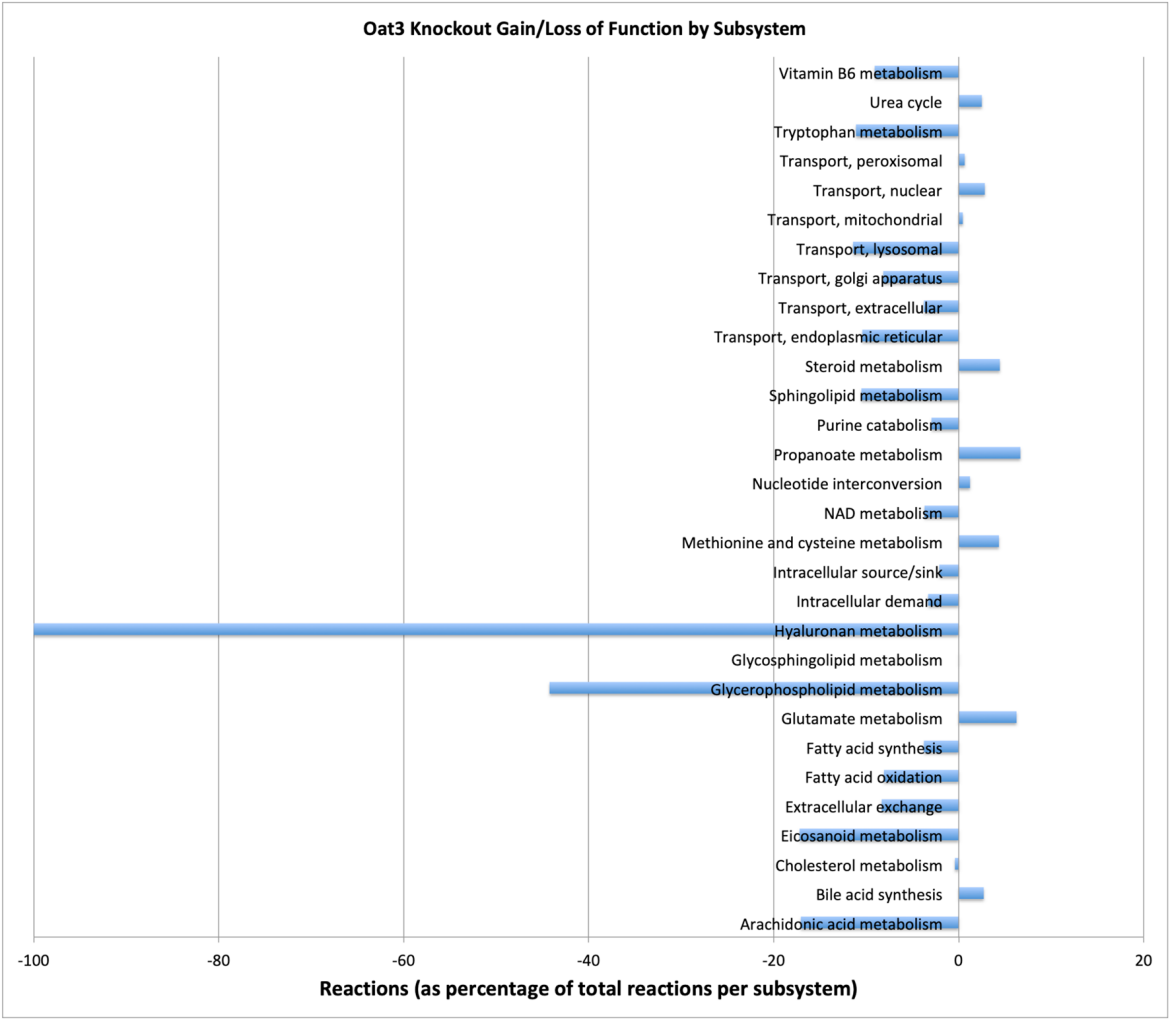
Changes in mutually exclusive reactions in the WT versus OAT3 KO models according to metabolic sub-systems given as a percentage of the total number of reactions present in each Recon3D sub-system. In the Oat3KO model, notable decreases are seen in hyaluronan and glycerophospholipid metabolism. It is important to also recognize that, even though the number of reactions in a particular sub-system may decrease, particular reactions within that sub-system may have an increase in mean flux (since anabolic and catabolic reactions may be grouped within a single sub-system).

Our initial analyses of the current reconstructed network were consistent with the notion that, while the OAT1 and OAT3 models share a large number of reactions, each OAT sub-type modulates many unique metabolic processes. Interestingly, when comparing WT and KO models and the number of reactions unique to each model, the Oat1KO had a relatively larger number of unique reactions compared to WT; in contrast, in the case of the Oat3KO, there were a relatively larger number of WT reactions compared to knockout (Fig. 3). One interpretation of this result is that compensation for loss of OAT1 necessitated a more pronounced metabolic compensatory response, which is consistent with the greater statistical significance of the numerous changes in metabolites in the knockout mice as well as the established role of OAT1 in energy metabolism in cell culture experiments^31^. The metabolic sub-systems corresponding to the unique reactions in WT and KO models for OAT1 and OAT3 (Fig. 3), respectively, are highlighted in Figs. 4 and 5. They include many reactions supported by in vitro and in vivo studies^22–24, 32, 33^.

**Figure 6:**
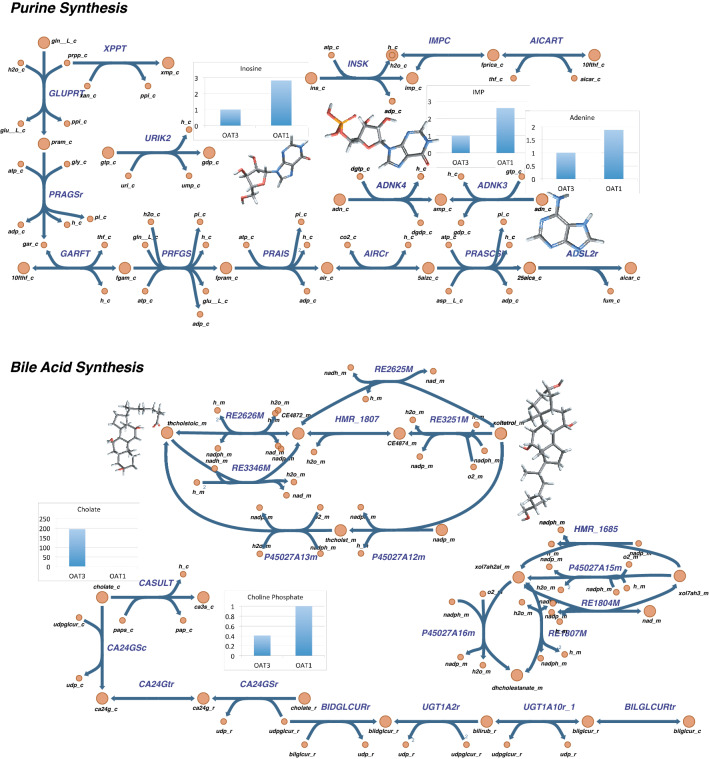
Highlight of reactions in Purine Synthesis (OAT1, Table 1) and Bile Acid Synthesis (OAT3, Table 1). *Purine Synthesis*: Reactions in the Purine Synthesis pathway that are not present in OAT1 WT models but are present in OAT1 KO are outlined. Fold change ratios of particular metabolites that were increased in OAT1 KO are presented next to their 3D bond-line molecular structures and include adenine, inosine, and IMP. *Bile Acid Synthesis*: A subset of the reactions that are present in the OAT3 knockout, but not the WT, are highlighted. Since the plasma concentration of choline is significantly higher in the OAT3 knockouts, the model pathways directly involving choline and choline-phospholipids were not increased (gray-shaded/opacified pathway at the base of the figure). Consistent with experimental data, however, pathways and metabolites with more complex, ring containing structures were identified in the OAT3 KO, including xoltetrol_m (3α,7α,12α,26-Tetrahydroxy-5β-cholestane) and thcholst_m (3α, 7α, 12α-Trihydroxy-5β-cholestan-26-al).

Two sub-systems that increased in number for *Oat1* and *Oat3* knockouts are schematized in Fig. 6 (Purine Synthesis for *Oat1* knockout and Bile Acid Synthesis for *Oat3* knockout). Purine metabolism intermediates, including inosine, inosine monophosphate (IMP), and adenine, that are also found to have increased plasma concentrations in *Oat1* knockouts, are highlighted. The Bile Acid Synthesis sub-system changes in the *Oat3* knockout are slightly more nuanced but appear consistent with the well-established role of *Oat3* in in vivo bile acid regulation^31^. The increased plasma choline concentration in the *Oat3* knockouts leaves less intracellular choline use for bile acid metabolism, leading to increased metabolism of other, multi-ring intermediates, such as 3 $\alpha$,7 $\alpha$,12 $\alpha$-Trihydroxy-5 $\beta$-cholestanoate.

### Organelle-based differences in metabolism

The role of plasma membrane multi-specific “drug” transporters in modulating intracellular and organelle-specific reactions has received limited attention to date. Nevertheless, there is strong in vitro experimental evidence that the OATs are intimately connected to the tricarboxylic acid (TCA) Cycle, and indeed, counter-transport of a TCA Cycle intermediate (generally assumed to be the dicarboxylic acid, alpha-ketoglutarate) is necessary for uptake of metabolites and drugs into the kidney proximal tubule cell^34^. Comparisons between the WT and KO models for the number of different metabolites for each of the 9 compartments (7 host-intracellular organelles, microbe-intracellular, and extracellular) were statistically significant for the knockouts of both *Oat1* and *Oat3* with Chi-square values of 103.9 (corresponding to p < 0.001) and 20.1 (corresponding to p < 0.01), respectively. Many of these metabolic processes are related to the endoplasmic reticulum (ER), a key site of metabolism of lipids, steroids, and xenobiotics (Fig. 3C). In contrast to the *Oat1* knockout changes, knockout of *Oat3* resulted in shifts (decreases) in Golgi, lysosome, and peroxisome metabolic processes (Fig. 3D).

### Metabolome and transcriptome analysis

(Fig. 2, Tier 2): System-wide changes from models incorporating transcriptomic and metabolomic data from the WT and Oat1 and Oat3 KO mice.

In order to further constrain the models and also expand the scope of the models by accounting for the plasma metabolomic measurements (i.e. the circulating metabolome), the metabolomic data from the four conditions (Oat1WT, Oat1KO, Oat3WT, Oat3KO) was mapped onto the model (Fig. 2; see Methods). Previous studies using early versions of reconstruction tools and very limited omics data focused on identifying novel metabolites that interacted with OAT transporters^22–24^. For the present analysis, given the much broader and deeper metabolomic coverage that has recently been achieved in the Oat1 and Oat3 knockouts, the interest was to assess more global alterations resulting from the OAT KO, including direct and indirect consequences from loss of *Oat1* and *Oat3*. Differentially active reactions achieving significance were identified by randomized sampling of the steady state flux solution space (Supplemental File 1). There were 189 and 142 significantly altered exchange metabolites for the WT versus the *Oat1* and *Oat3* KO conditions, respectively (104 shared metabolites).

### Vitamin and micronutrient shifts

**Figure 7:**
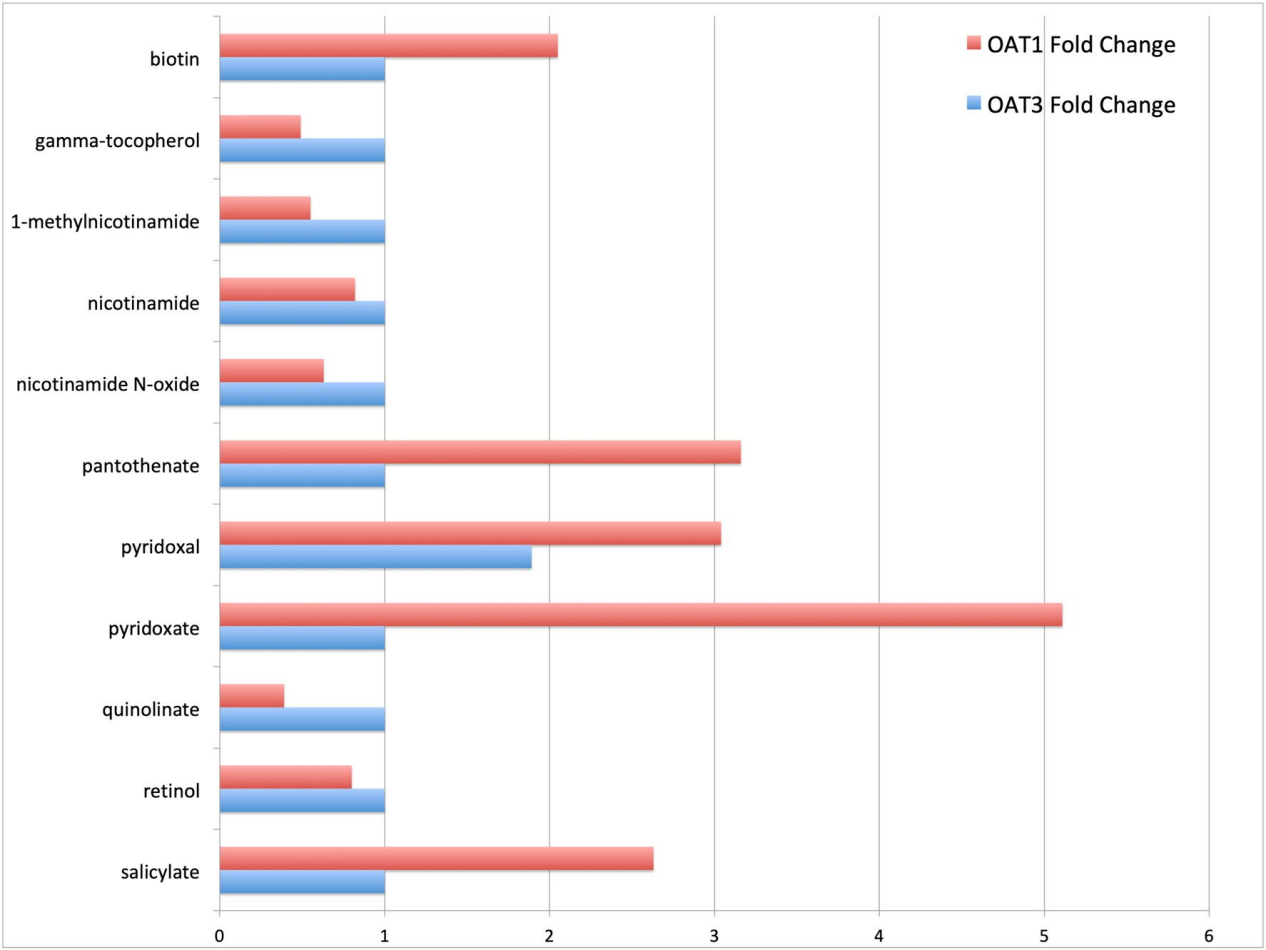
Changes in vitamin concentrations in OAT1 and OAT3. Relative ratios (KO/WT) of vitamin and vitamin precursor concentrations for the OAT1 and OAT3 knockouts. The changes in the availability and transport of these metabolites in the context of the altered microbiome interactions as well as organ-organ interactions, such as the kidney-liver-colon axis, suggests there may be nutritional consequences resulting from loss of OAT expression.

Among the notable total changes in the OAT knockouts are the shifts in vitamin and their precursor metabolites in the plasma; many of these changes occur in the *Oat1* knockouts^32^. Both the *Oat1* and *Oat3* knockouts have increased plasma pyridoxal concentrations. However, the Oat1 knockouts have increases in numerous other vitamins including biotin, pantothenate, pyridoxate, and salicylate (Fig. 7). Notable decreases include tocopherol, nicotinamide, quinolinate, and retinol. Additionally, changes in the glutathione and associated redox pools were observed, notable for increased ophthalmate in both *Oat1* and *Oat3* knockouts; increased *S*-methylglutathione was seen only in *Oat3* knockouts and increased oxidized glutathione and cysteine-glutathione disulfide only in *Oat1* knockouts.

### Co-set analysis identifies host-microbiome interactions

**Figure 8:**
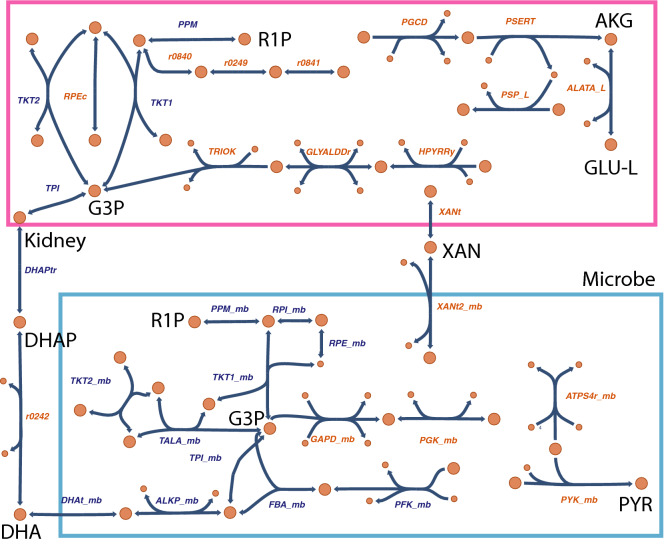
Kidney-microbiome interactions revealed by analysis of reaction co-sets connects host (kidney) and gut microbial metabolic pathways via transport. Reaction co-sets provide a means of identifying functionally correlated pathways by delineation of groups of reactions that are linked together based upon flux correlations^77^. The kidney-microbiome steady state flux space was interrogated in order to identify reaction co-sets involving the host as well as the microbiome, reflected as changes in the plasma metabolite concentrations. The illustrated central metabolic pathways centering around hexose and pentose sugar metabolism provide direct links between the host and microbiome. Links between glycolysis and the non-oxidative pentose phosphate pathway are directly coupled via inter-organismal (host-microbiome) transport of dihydroxyacetone phosphate and dihydroxyacetone. Other metabolites such as xanthine and L-alanine are present in some but not all of the co-sets for the four models (Oat1WT, Oat1KO, Oat3WT, Oat3KO), reflecting the condition/context dependence of the co-sets. Reactions with blue names are present in all four models (reactions with red names are present in one, two, or three models). Abbreviations are as follows, with those enzymes and transporters present in the microbiome as opposed to the host indicated as “x_mb (microbiome)”: *ALATA_L* l-alanine transaminase, *PSERT* phosphoserine transaminase, *PGCD* phosphoglycerate dehydrogenase, *PSP_L* phosphoserine phosphatase (l-serine), *r0841* RU5P ER to cytosol transport, *r0249* d-ribose-5-phosphate ketol-isomerase, *r0840* R5P ER to cytosol transport, *PPM* phosphopentomutase, TKT1: Transketolase, *RPEc* ribulose 5-phosphate 3-epimerase, *TKT2* transketolase, *TRIOK* triokinase, *GLYALDDr* d-Glyceraldehyde dehydrogenase, *HPYRRy* hydroxypyruvate reductase (NADPH), *TPI* triose-phosphate isomerase, *XANt* xanthine reversible transport, *r0242* glycerone phosphate phosphohydrolase, *DHAPtr* dihydroxyacetone phosphate transport, *XANt2_mb* xanthine transport (microbiome), *PPM_mb* phosphopentomutase (microbiome), *RPI_mb* ribose phosphate isomerase (microbiome), *RPE_mb* ribulose 5-phosphate 3-epimerase (microbiome), *TKT1_mb* transketolase (microbiome), *TKT2_mb* transketolase (microbiome), *TALA_mb* transaldolase (microbiome), *TPI_mb* triose-phosphate isomerase (microbiome), *GAPD_mb* glyceraldehyde-3-phosphate dehydrogenase (microbiome), *PGK_mb* phosphoglycerate kinase (microbiome), *ATPS4r_mb* ATP synthase (microbiome), *DHAt_mb* dihydroxyacetone transport (microbiome), *ALKP_mb* alkaline phosphatase (microbiome), FBA_mb: Fructose-bisphosphate aldolase (microbiome), *PFK_mb* phosphofructokinase (microbiome), *PYK_mb* pyruvate kinase (microbiome), *AKG* alpha ketoglutarate, *GLU-L* l-glutamate, *R1P* ribose-1-phosphate, *G3P* glyceraldehyde 3-phosphate, *XAN* xanthine, *DHAP* dihydroxyacetone phosphate, *DHA* dihydroxyacetone, *PYR* pyruvate.

Many gut-derived products entering the circulation are organic anions that are transported by OATs into kidney proximal tubule cells whereupon they are eliminated^22, 29, 32^ and/or affect metabolism and signaling via, for example, nuclear receptors within kidney cells^35, 36^. Reaction co-sets can provide insights into these complex metabolic pathways by analyzing functionally connected sets of reactions. Not surprisingly, the largest co-sets for all four models (Oat1WT, Oat1KO, Oat3WT, Oat3KO) were driven by reactions related to production of microbial biomass. However, uniformly across all of the models, the second largest co-set involved central metabolites in host and microbial metabolism (Fig. 8). While some metabolites are specific to microbes, other metabolites in evolutionarily conserved pathways, may be disrupted in the host and/or the microbiome. R1P, G3P, AKG, PYR, GLU-L were found to be altered in the plasma metabolomic data, however these measurements alone provided no insight into inter-dependenices or whether the changes resulted from the host or the microbiome. The host-microbial co-set depicted in Fig. 8 highlights the mechanistic connectivity between some of these shared metabolites within the kidney and microbe. Further the co-set revealed that the intracellular changes could be transmitted/communicated between the kidney and microbe via dihydroxyacetone phosphate (DHAP), dihydroxyacetone (DHA), and XAN. Thus, DHA appears to be a critical link between sugar metabolism in the host and microbe (glycolysis and non-oxidative pentose sugar metabolism), in addition to xanthine and alanine.

**Figure 9:**
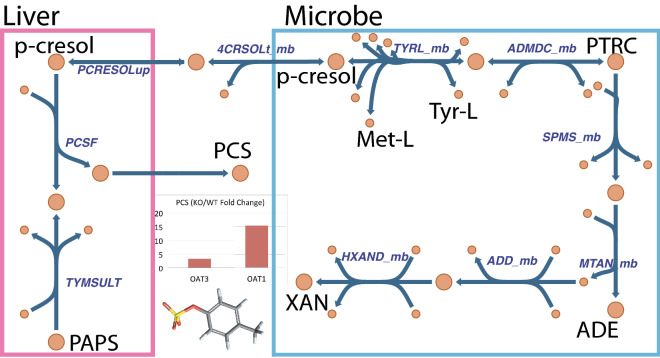
Analysis of liver-microbiome interactions connects host (liver) and gut microbial metabolic pathways via transport of *p*-cresol. There were significant changes in some metabolites detected in the serum that were specific to microbial biosynthesis pathways. This schematic illustration of parts of L-methionine and L-tyrosine pathways connecting host and microbiome metabolism via *p*-cresol, a metabolite that cannot be produced by mice or humans, was observed to be significantly altered in OAT1 as well as OAT3 knockouts. This map reveals the hepatic-microbial linkage for *p*-cresol metabolism. Tyrosine lyase acting upon L-tyrosine and *S*-adenosyl-L-methionine yields L-methionine and *p*-cresol in the microbe. In turn, hepatocytes take up *p*-cresol, whereupon it is sulfated, and then secrete as *p*-cresol sulfate for subsequent elimination by the kidney through the OATs. Thus, this is an example of inter-organismal and inter-organ communication via the gut microbe-host liver-host kidney. Abbreviations are as follows, with those enzymes and transporters present in the microbiome as opposed to the host indicated as “x_mb (microbiome)”: *PCSF* *p*-cresol sulfation, *TYMSULT* Tyramine sulfotransferase, *PCRESOLup* *p*-cresol uptake, *4CRSOLt_mb* *p*-cresol transport (microbiome), *TYRL_mb* Tyrosine lyase (microbiome), *ADMDC_mb* Adenosylmethionine decarboxylase (microbiome), *SPMS_mb* Spermidine synthase (microbiome), *MTAN_mb* methylthioadenosine nucleosidase (microbiome), *ADD_mb* adenine deaminase (microbiome), *HXAND_mb* hypoxanthine dehydrogenase (microbiome), *PAPS* 3-phosphoadenylyl sulfate, *PCS* *p*-cresol sulfate, *Met-L* l-methionine, *Tyr-L* l -tyrosine, *ADE* adenine, *XAN* xanthine.

Another example among these significantly altered metabolites linking host-microbe metabolism is the metabolism and handling of *p*-cresol, which turns out to be similar to the experimentally well-supported and paradigmatic example of gut microbe-derived OAT substrate indoxyl sulfate^35–38^—though, in this case, revealed by analysis of reaction co-sets. *p*-cresol is generated in the microbiome during conversion of L-tyrosine to L-methionine and subsequently absorbed by the host. *p*-cresol is, in turn, sulfated by hepatocytes and secreted from the liver (Fig. 9). The subsequent excretion by the kidney completes the gut microbiome-liver-kidney axis. This inter-organismal and inter-organ pathway has been described and confirmed in the experimental literature^5, 28, 29, 39, 40^. Furthermore, the recapitulation of this pathway explicitly with the models raised the question of whether other inter-organ interactions could also be identified with these data.

### Inter-organ interactions

When we further examined the results of metabolomics-constrained genome-scale analyses (including host-microbe interactions) from the two transporter KO mice and two WT mice, we found many reactions relating to tissue-specific functions of other organs. This suggested that the new metabolomics-constrained host-microbe models, which already revealed intracellular and intra-organelle reactions, also included reactions spanning multiple organs.

Tissue-Specific Expression Analysis (TSEA)^41^ was used as an independently validated approach for identifying tissue-specific subsets of genes for kidney, liver, brain, skin, adipose, and colon (see “Methods”). The network reconstruction was used to define corresponding metabolic functions for these gene sets. The metabolic demands for each of these tissues compete for the availability of different metabolites depending upon reactions that are tissue-specific, or relatively so. Interestingly, correlations between any two tissues are relatively poor, suggesting that our approach captures key aspects of organ-selective metabolic processes (in the context of the host-microbe unit) that are dependent upon *Oat1*, *Oat3*, or both.

**Figure 10:**
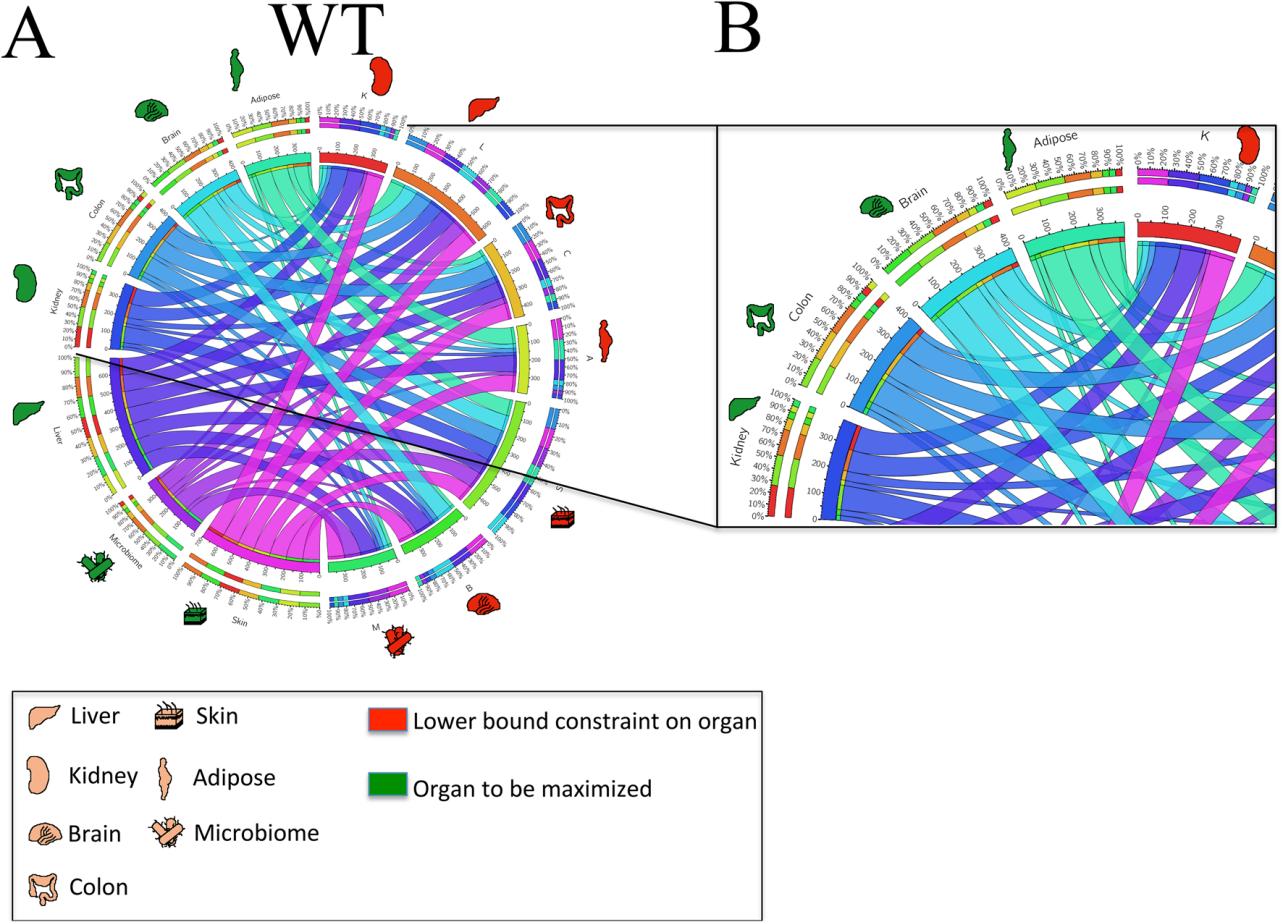

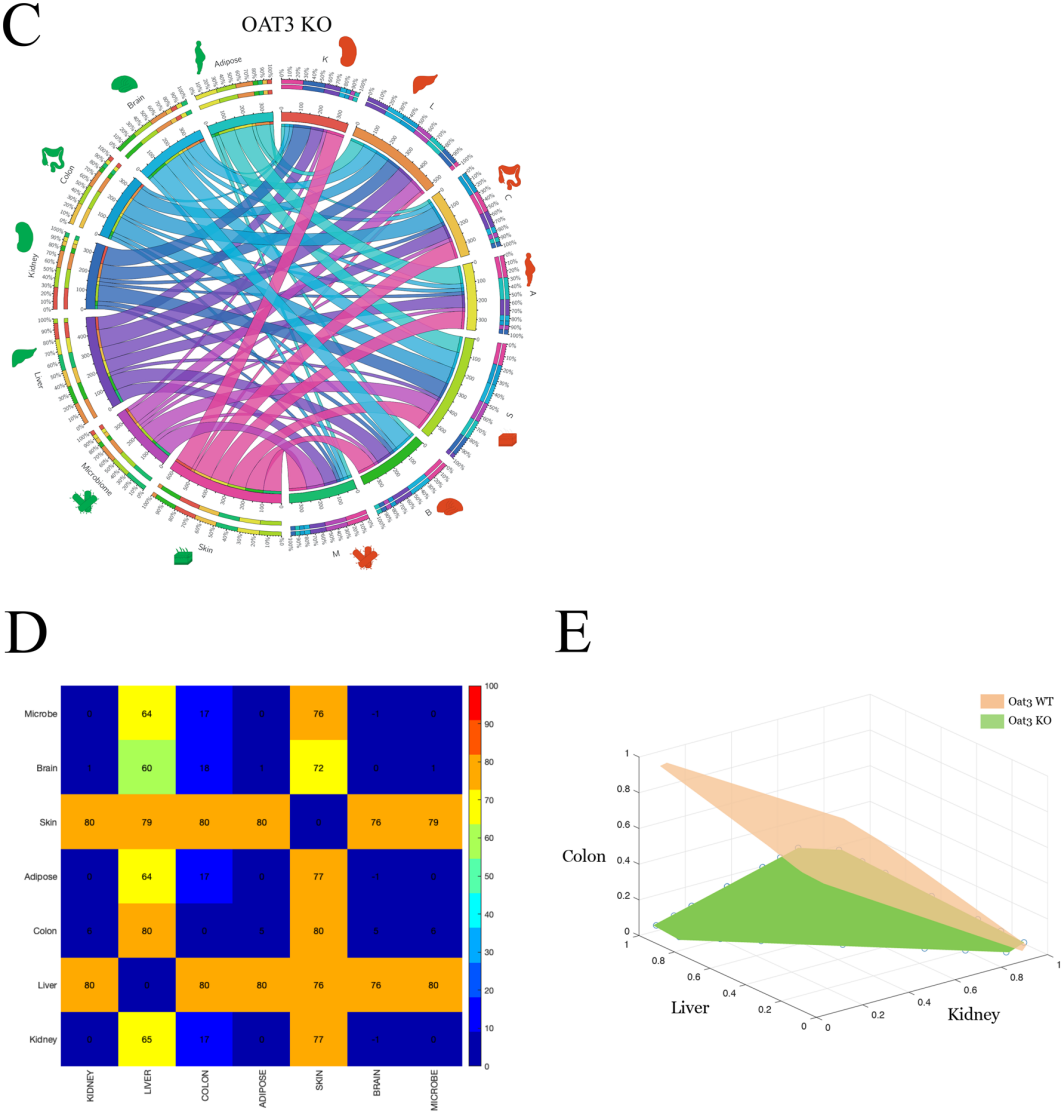
Organ-organ interaction and metabolic interdependence for OAT3 WT and KO models. The models were analyzed to assess the influence that different organ metabolic objectives had on one another and whether these changed with the loss of OAT function. The Circos plots illustrate direct pairwise interactions and the consequences of reaching a particular organ’s metabolic objective while being directly constrained by a different organ. The *red shaded organ icons* can be viewed as the output constraining effect, and the *green shaded organ icons* as the input constraining effect (‘constraining effect’ refers to a limit on a particular reaction flux as a result of competing substrates for a different metabolic reaction). The *ribbon width* of each organ to its self-counterpart (i.e. red colon to green colon) serves as the ‘reference’ comparison for other organ-organ interactions. Similar ribbon widths reflect relative independence of the optimized organ. Conversely a smaller width indicates a constraining effect on the optimized organ metabolic objective. (**A**) WT Circos plot of organ-organ interactions with ribbon width corresponding to the maximum percentage of an organ’s metabolic objective while maintaining 80% of maximum for another organ. The majority of the ribbons are symmetric with similar widths. Notably there is a constraining effect of the kidney on the colon, microbiome, and brain metabolic objectives. (**B**) Zoomed in view of a section of the WT Circos plot. The outer ring scales are percentages relative to the summed total independently optimized organ objectives. The inner ring is the summed total normalized to a total value of 400. The width of the kidney-kidney ribbon serves as the reference. Note that the kidney → microbiome, kidney → colon, and kidney → brain ribbons (green → red for each respective case) are markedly thinner (approximately 1/10 each), indicating metabolic competition for each of these organs. (**C**) Knockout Circos plot of organ-organ interactions with ribbon width corresponding to the maximum percentage of an organ’s metabolic objective while maintaining 80% of maximum for another organ. Relative comparison between the WT (**A**,**B**) and KO (**C**) indicate changes in the inter-organ metabolic demands resulting from loss of OAT3 transport function. The kidney-colon-microbiome axis has been described, but there is a constraining effect of liver metabolism on multiple organs including the kidney, brain, and microbiome. In Supplemental Fig. 3, a similar analysis is presented for WT versus *Oat1* KO. (**D**) Difference (in percentages) between the WT and KO Circos plots. The quantitative percentage differences between the organ-organ interactions for WT (**A**,**B**) and KO (**C**) in panel (**D**) provide an objective assessment of the changes in the inter-organ constraints due to loss of OAT3. The most prominent changes are seen for the liver, colon, and skin. (**E**) Kidney-liver-colon flux cone. The 3D flux phase plane depicts the trade-offs among three competing organ objectives, the kidney, liver, and colon. It is interesting to note that, in comparing the WT and KO, although the liver-kidney slope is curtailed, the metabolic flexibility for the colon is severely restricted. Legend: Labels for the different organ icons are described. *Red shading* reflects the organ that has been constrained with a lower bound equal to 80% of its maximum value. *Green shading* demarcates the organ metabolic objective that is optimized. Abbreviations (corresponding to the organ icons): *L* liver, *K* kidney, *B* brain, *S* skin, *A* adipose, *C* colon, *MB* microbiome.

We considered pairwise interactions among the six different organs plus the microbiome in order to determine whether an increased metabolic demand for one tissue affects (or doesn’t affect) the metabolic capabilities of a different organ. The relative interactions for the WT and KO mice were then compared in order to provide an assessment of the changes (if any) between different organs that result from transporter loss of function in the *Oat1* or *Oat3* knockout mouse. For each of the different organs and microbiome, the metabolic demand for each tissue was optimized while setting a non-zero demand for a different organ or microbiome (see “Methods”). Crucially, by this approach, every pairwise influence of a particular organ on another organ can be assessed (Fig. 10).

The Circos plots of the optimized metabolic organ objectives (Fig. 10A,B for *Oat3* WT and KO, respectively) provide a visual depiction of the metabolic demands of one organ on another. The width of each ribbon relative to itself (e.g. red brain to green brain or red kidney to green kidney) represents the ‘reference’ metabolic demand for that organ. The width of the self-organ ribbons in comparison to the inter-organ ribbons (e.g. red brain to green kidney, red kidney to green liver, etc.) represents the competitive metabolic demand of the other (green) organ. Differences in the weightings were then compared (as percentages) between the wildtype versus knockout models.

From these Circos plots, one can immediately appreciate the asymmetric metabolic demands of different organs on one another qualitatively and as quantitative percentage differences (Fig. 10C). Moreover, in assessing the kidney-liver-colon axis, it is interesting that across a range of varying metabolic demands, the metabolic flexibility of the colon is most severely restricted as a consequence of *Oat3* KO (Fig. 10D).

Interestingly, the organ-organ interaction difference maps are asymmetric for the *Oat1* (Supplemental Figs. 2 and 3) and *Oat3* WT versus KO comparisons (Fig. 9). This supports the interpretation that the organ-organ interactions are not static and can potentially shift depending on changes in substrate availability and metabolic stresses on particular organs. Additionally, the WT versus KO organ-organ interaction plots for *Oat1* indicate the largest alterations for the liver and colon (followed by adipose and skin), whereas for the *Oat3* the largest alterations occurred with the liver and skin (followed by the colon). Finally, since these interactions are occurring simultaneously and dynamically, the conceptual model of a hierarchical axis gives way to a structured web of interactions.

**Figure 11:**
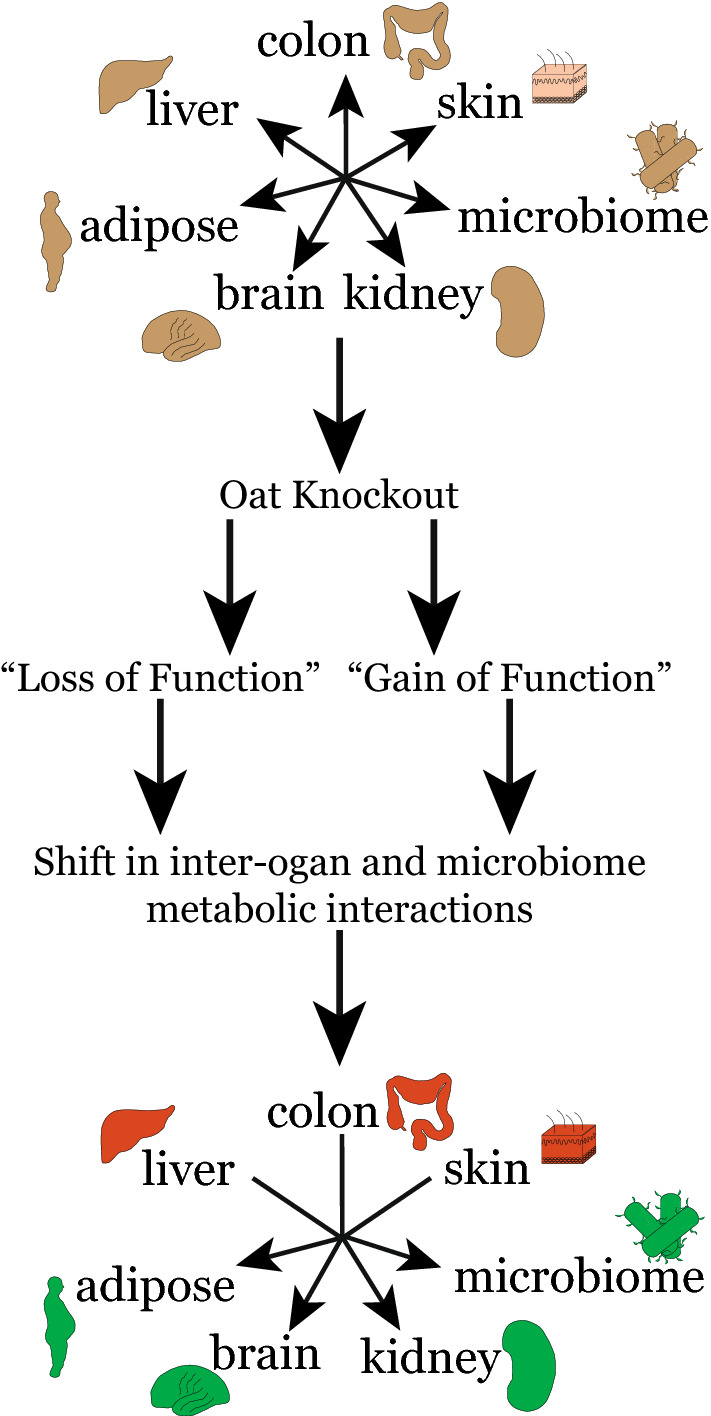
OAT KO loss and gain of function. The constraint on cellular small metabolite transport capabilities due knockout of *Oat1* or *Oat3* can indirectly lead to apparent ‘gain of functions’ via altered substrate availability to different organs; this may provide a partial explanation for the observed remote organ effects. Liver, colon, and skin are noted to also exert constraining effects on metabolic objectives of other organs.

Collectively, we see the consequences of OAT influence on systemic metabolism extending beyond the kidney-microbiome axis and involving multiple organs. Our results suggest that interactions between the liver, colon, and skin with the other organs are most prominently affected by loss of OAT function (Fig. 11). The interactions between renal OATs and skin metabolism may seem unexpected and has not been studied in any detail. Nevertheless, it is important to note that it is a large complex organ that is a key site of Vitamin D, fatty acid, organic acid, and melatonin/amino acid metabolism, and skin has its own sets of microbiomes^42–44^.

## Discussion

This reconstruction provides the first multi-scale representation of how multiple drug transporters regulate metabolism across organelles, cells, organs, multi-organ axes (e.g. gut-liver-kidney), and organisms (e.g. gut microbes-body). Many interesting insights emerge. For instance, to compensate for the loss of transport by a plasma membrane OAT, cells in the kidney alter a large set of reactions occurring in certain organelles. More generally, the reconstruction supports the RSST, which seeks to explain the role of "drug" transporters and other proteins—many of which are also involved in the drug absorption, distribution, metabolism and elimination (ADME) of small molecule drugs—in inter-organ and inter-organismal communication across multiple scales (organelle to multi-organism)^3, 5, 10, 12^. The RSST is discussed further below.

Considering both OAT1 and OAT3 together is particularly interesting and important from a physiological perspective. These two "drug" transporters are, together, the main route of renal handling of a very large class of clinically-relevant small molecules: organic anions. One of the main roles of the kidney is the elimination of small molecule drugs and toxins (exogenous and endogenous), as well as numerous by-products of metabolism, including uremic toxins of CKD originating from gut microbes. Many of these gut-microbe small molecules are organic anions with molecular weights on the order of 100–1000 Da known to be transported by OATs in the kidney proximal tubule.

While OAT1 and OAT3 are among the seven drug transporters initially focused upon by regulatory agencies because their immense pharmaceutical and pharmacokinetic importance^5, 45, 46^, a growing amount of evidence indicates an essential role for OATs in many aspects of endogenous physiology^22–24, 29, 47^. They and other closely related SLC22 family transporters are expressed early in organ development^39, 40^, and OATs and other SLC22 transporters are evolutionarily conserved, with orthologous and/or highly homologous genes found in mouse, zebrafish, fly, and worm^48^. Indeed, many family members in fly are essential for development of the embryo, or in adults, recovery from oxidative stress^33^. In zebrafish, OATs appear to have endogenous functions similar to mammals^49^. There continues to be growing evidence for the direct relevance of mouse OATs and humans^48, 50, 51^, further supporting the important role of murine experimental models. While the murine knockouts of *Oat1* and *Oat3* have the predicted pharmacological and toxicological phenotypes, the application of metabolomics has radically changed our understanding of OAT function^23, 24, 30, 45, 52, 53^. Much of this wet lab experimental data comes from our research group.

In addition, numerous in vitro and in vivo experiments carried out by multiple groups, including ours, have revealed connections of OAT1 and OAT3 to the transport of gut microbe-derived products such as hippurate and *p*-cresol sulfate^54–56^, intracellular molecules such as TCA cycle intermediates^9^, and links between Phase 1 and Phase 2 enzymatic modifications of endogenous molecules by the liver (e.g. glucuronidation, sulfation) and secretion by the proximal tubule of the kidney^57^. Co-expression networks including SLC and ABC transporters support the existence of endogenous networks linking the physiology of the gut, liver, and kidney to many multi-specific, oligo-specific and mono-specific transporters with SLC22 transporters (including OATs, OCTs, and OCTNs) forming key hubs in the transporter network by several network metrics^29^.

Nevertheless, a formal analysis of these connections across multiple scales (organelles, cells, organs, organisms) has been lacking until now, leaving much of the aforementioned experimental data piecemeal and unconnected. Through analysis of multiple omics datasets from the *Oat1* and *Oat3* knockouts using a host-microbiome metabolic reconstruction, we have shown, to our knowledge for the first time, alterations in biochemical reactions involving two major multi-specific “drug” transporters affecting intra-cellular, inter-organ, and inter-organismal metabolic capabilities. These endogenous biochemical reactions are, at various scales, dependent upon OAT1, OAT3, or both.

We used TSEA as a reconstruction-independent means to define tissue-specific functions and then analyzed the metabolomic data with different organ objectives. Indeed, it is interesting to note how one or the other transporter impacts certain non-renal organs similarly and differently (Fig. 10) as well as their differential impact on cellular and organelle (e.g. endoplasmic reticulum) reactions (Fig. 3). Thus, through a novel linkage of systems biochemical methods, transcriptomic, and metabolomic data from WT versus *Oat1* and *Oat3* KO mice, new insight is provided into the local and systemic effects of OATs, including connections to relevant aspects of host metabolism dependent upon the metabolism in the gut microbiome.

The implications for understanding the scope of drug transporter biology, as opposed to more narrowly-defined pharmacokinetics analyses, are far-ranging and raises many new questions for research in this field. The metabolomic and transcriptomic measurements in conjunction with the systems analysis implicating altered interactions of multiple organs in the context of loss of OAT functions may have significant implications for understanding triggers for multi-organ failure when patients are administered multiple therapeutic drugs including probenecid, antivirals, and penicillin-based antibiotics, for example, in the setting of compromised renal or hepatic function.

One of the most unexpected findings of this study is the connection between kidney OATs and the skin. We emphasize again a significant and largely unappreciated role of skin metabolism relating to endogenous metabolites and drugs. A growing number of experimental and clinical studies, however, indicate a significant role of skin as a drug metabolizing organ^58^. Its role in systemic metabolism is only beginning to be explored. Another interesting and unexpected finding was the shift in glutathione metabolism in OAT KO models. Interestingly, ophthalmate has been directly linked to biochemical aging of cells with associated decreased glutathione availability^59^. The ability to maintain redox pools and to respond to exogenous and endogenous redox loads is a core, essential function of cellular metabolism. An impaired ability to tolerate redox stresses, as suggested by the OAT KO models, may have practical implications in our understanding of ‘off-target’ effects of drugs interacting with these transporters. Future studies to further evaluate these mechanisms, as well as understanding regulation of different sub-pathways in *Oat1* versus *Oat3* KO (e.g. increased cysteine-glutathione disulfide versus *S*-methylglutathione, respectively) may expand our understanding of metabolic regulation of redox states.

A critical challenge with analyzing OATs and other multi-specific (e.g. MRP2 or ABCC2, OATP1B3 or SLCO1B1) and oligo-specific transporters (e.g. organic cation/carnitine transporter 2 (OCTN2 or SLC22A5), multidrug and toxin extrusion protein 2 (MATE2 or SLC47A2)), most of which are usually considered drug transporters, is the spectrum of their transport capabilities not only for drugs but also endogenous molecules (e.g. short chain fatty acids, cyclic nucleotides, antioxidants, bile acids)–resulting in local and systemic effects secondary to their loss of function.

The Remote Sensing and Signaling Theory (RSST)^60^ posits that key multi-, oligo-, and monospecific transporters, "drug" metabolizing enzymes, and nuclear receptors mediate communication via small molecules (e.g. metabolites, signaling molecules, antioxidants) between organs and organisms (e.g. host-microbiome). This ultimately coordinates the shifts in the availability of small molecules with "high informational content" across different tissues and fluid compartments in the body^3^. By and large consistent with the RSST, the systems approach employed here using transcriptomic and broad plasma metabolomic coverage takes steps towards analyzing how metabolic signals are transmitted across many scales including organelles, cells, organs, the mammalian organism (whole body), and the interfacing microbiome. More complete formulations of the RSST—as well as the experimental evidence behind the theory and applications to human disease—have been presented elsewhere^3, 5, 8, 10, 47, 61^.

OAT1 and OAT3 are but two of the > 500 proteins (consisting of transporters, DMEs, nuclear receptors and other proteins) that appear to be part of a Remote Sensing and Signaling protein network regulating small molecule homeostasis^45, 61^. The SLC22 transporter family, of which OAT1 and OAT3 are among the best-known members, is a key hub in the Remote Sensing and Signaling protein network^45^. It would be particularly interesting, provided similar omics data becomes available, to perform metabolic reconstructions of members of other transporter, DME, and nuclear receptor families that are hubs in the Remote Sensing and Signaling protein network (e.g. BCRP, MRP2, HNF4a). This would give a more complete portrait of how these multi-, oligo- and monospecific transporters, enzymes and regulatory proteins affect different aspects of metabolism across multiple scales.

The context-specific genome-scale reconstructions that we present here were based on: (1) the transcriptomic alterations in the *Oat1* and *Oat3* knockout mice; (2) connecting them to bacterial metabolic reconstructions; and then 3) constraining by knockout metabolomics (as done here). The resulting reconstruction provides key and novel insights into how the system has responded, in terms of metabolic capabilities, to the loss of function of one or both OATs. However, one challenge with high-throughput profiling of complex systems with dynamically interacting organelles, cells, organs, and organisms, is that the direction of causality cannot be immediately inferred. We acknowledge that this multi-scale model remains incomplete and simplified. Currently, it is difficult to perform rigorously controlled ex vivo multi-organ plus microbiome experiments that authentically reproduce in vivo inter-organ and inter-organismal cross-talk with simultaneous omics profiling at the many scales necessary to fully evaluate the model. However, this should be possible within a few years. Such experiments should be able to help refine the model as well as add complexity in a step-by-step fashion that is not easily done with in vivo experimentation even with multi-omics readouts. Then predictions of such multi-scale metabolic reconstructions can be further refined based on dynamic data based on ex vivo model perturbations in light of comparable in vivo perturbations in well-established physiological and pathophysiological animal models.

Importantly, through the use of organ-specific transcriptomics and plasma (systemic) metabolomics, the resulting models are a step towards reconciling genome-scale metabolic networks with ADME models. Thus, they provide a novel, rigorous, and quantitative avenue for relating the systems biology of metabolism to quantitative systems pharmacology (QSP) and physiologically-based pharmacokinetics^14, 61, 62^. As such, the work also represents a foundational approach to understanding transporter-based drug-metabolite interactions beyond a single transporter, beyond a single organ, and—given that it considers the microbiome—beyond a single organism. The implications are far-ranging in that they both provide a basis for contextualizing previous in vivo studies^51, 63–66^ and also suggest the types of detailed analyses that will need to be done in future studies in model organisms and humans to more clearly define the multi-scale (i.e. intra-organelle, intra-cellular, inter-organ, and inter-organism) impact of small molecule drugs and drug transporter perturbations on metabolism and physiology.

Ideally, we would like to integrate human metabolism with many more species of gut microbes; we would also like to have omics data at many scales from multiple organs and cell types for genome-scale metabolic reconstructions. We have used to advantage the fact that OAT1 and OAT3—while experimentally demonstrated to regulate or modulate many local and systemic metabolic pathways and shown to be key determinants of plasma levels of gut microbe-derived molecules^7, 53, 56^—have 90–95 percent of their total expression in a single organ, the kidney. A major function of the kidney is to mediate, in large part via the activity of OAT1 and OAT3, the elimination of small organic anions. Thus, the quantitative data on levels of bacterial-derived metabolites made it possible to integrate a microbiome model into the multi-scale reconstruction. While we were able to incorporate basic interactions, future iterations may further build upon this as more extensive data becomes available.

This overwhelming expression of the OATs, the main drug transporters in the single organ responsible for the elimination of many common drugs (e.g. antibiotics, NSAIDs, HIV antivirals), is in marked contrast to many other multi-specific drug transporters such as p-glycoprotein (ABCB1), breast cancer resistance protein (BCRP, ABCG2), multidrug resistance related protein 2 (MRP2, ABCC2), which are expressed in a number of organs involved in drug handling such as the gut, liver, and kidney. OAT1 and OAT3 are, however, not expressed in the adult gut and adult liver.

It is important to re-emphasize that the vast preponderance of expression of the OATs in a single organ seems to have been an advantage for our reconstruction in that we did not have to reconcile transcriptomics data from multiple organs, as might be necessary in the case of well-known multi-specific ABC drug transporters expressed broadly across many organs; in this latter case of major expression in many organs, the appropriate methodological approach is also far less clear. Given the aforementioned expression patterns, OAT knockout transcriptomics data from the kidney, constrained by plasma metabolomics, seems likely to yield a useful multiscale reconstruction of drug transporter-mediated metabolism. This view is strongly supported by the considerable in vitro and in vivo wet lab data already discussed. As more datasets are generated, we anticipate that the model predictions will improve with greater specificity, although it is expected that the general observations from the current simulations will be refined rather than be radically altered.

In the future, exposing and exploring the potential to identify organ-specific side-effects of renal eliminated drugs with multi-organ metabolic reconstructions (MOMR) may become critical for understanding the extent of direct and indirect drug-metabolite interactions. This is likely to be particularly important in the care of patients, who have pre-existing organ compromise (e.g. CKD) and who receive multiple medications, as these can conceivably lead to iatrogenic precipitation of multi-organ dysfunction.

## Methods

### Transcriptomic profiling

All experimental protocols involving the use of animals were approved by the UC San Diego Institutional Animal Care and Use Committee (IACUC) and consistent with ARRIVE guidelines (arriveguidelines.org). All animals were handled in accordance with the Institutional Guidelines on the Use of Live Animals for Research. Adult WT, *Oat1* KO, and *Oat3* KO males were housed separately under a 12-h light–dark cycle and were provided ad libitum access to food and water. Serum was extracted from the whole blood. These animals have been described in previous publications^22, 28^.

### Metabolomic profiling

Individual, unpooled samples were measured by the Metabolon analytical system (Metabolon, Inc., Durham, NC). Samples were prepared and subjected to ultrahigh performance liquid chromatography-tandem mass spectroscopy (UPLC-MS/MS) utilizing an ACQUITY ultra-performance liquid chromatography (UPLC) (Waters, Milford, MA) and a Q-Exactive high resolution/accurate mass spectrometer interfaced with a heated electrospray ionization (HESI-II) source and Orbitrap mass analyzer operated at 35,000 mass resolution (Thermo Scientific, Waltham, MA). Raw data was extracted, peak-identified and QC processed using Metabolon’s hardware and software^60, 61^. Two-way Analysis of Variance (ANOVA) testing was used to calculate the p-values and the metabolites that were selected for display in figures and tables had either: (1) a fold change ≥ 1.2 with a p-value ≤ 0.05; or (2) a fold change ≥ 2.0 with a p-value ≤ 0.1 in at least one of the various comparisons (e.g., Oat1KO vs WT; Oat3KO vs WT) (Supplemental File 1). Updated, expanded plasma profiling was performed as described^30^.

### Modeling and analysis

Multiple genome-scale metabolic reconstructions were used to construct a scaffold for data mapping and simulation including Recon3D and multiple microbial reconstructions. The “representative” microbiome microbe model (MB) was constructed starting with the *Bacillus subtilis* (iYO844)^67^ metabolic network reconstruction with incorporation of pathways from *Lactococcus lactis* MG1363 (iNF517)^68^, *Escherichia coli* K-12 MG1655 (iAF1260)^69^, *Escherichia coli* ETEC H10407 (iETEC_1333)^70^, and *Escherichia coli* (iML1515)^71^. In particular, 2-hydroxy-3-methyl pentanoate metabolism including biosynthesis from S-3-Methyl-2-oxopentanoate and transport were incorporated from iNF517, secretion of *S*-methyl-l-methionine from iAF1260, glutarate metabolism pathway from iML1515, and tyrosine metabolism from iETEC_1333. The role of the microbiome is to represent a distinct ‘organ’ that interacts with the kidney and other organs, and most importantly, accounts for some of the metabolites that are not endogenously produced by murine organs.

The GIMME algorithm maps gene expression data as reaction weightings and calculates context-specific subnetworks to achieve particular metabolic objectives using FBA^15^. These different networks are then compared to calculate a ‘consistency score’ that is minimized to optimize alignment of the resultant network with the gene expression data.

For the metabolic network with m metabolites, n reactions and corresponding stoichiometric matrix, $S\in {\mathbb{R}}^{mxn}$, reaction flux vector, $v\in {\mathbb{R}}^{n}$ (and corresponding upper and lower bounds, ${v}^{u} and {v}^{l}$ respectively), and reaction objective vector, $c\in {\mathbb{R}}^{n}$,

$$\mathrm{min}({c}^{T}\cdot \left|v\right|)$$

subject to

$$S\cdot v=0$$

$${v}^{l}\le v\le {v}^{u}$$

with $c= {x}_{cutoff}-x$ for $c>0$, else $c=0$. Conversion of the above to a linear programming problem is performed by constructing a convex null space by redefining the flux vector such that, $v={v}^{+}-{v}^{-}, 0\le {v}^{+}\le {v}^{u}\,\, \mathrm{and}\,\, 0\le {v}^{-}\le {-v}^{l}$, as described in^15^ and implemented in^72^.

The mouse transcriptomic data were mapped to human orthologs using the NCBI Homologene database (accessed October 27, 2019). Since transcript expression levels and fluxes are qualitatively associated, but generally not quantitatively correlated, the GIMME algorithm was used to apply context-specification with the transcriptomic data for WT and KO model construction. As described previously, transcriptomic data were incorporated based on present/absent (P/A) calls using Affymetrix Microarray Suite Version 5.0^22^. A minimum of 3 microarray datasets were included for each condition (WT and KO) and were analyzed separately; for a gene to be considered present, it had to be present in at least 2 of 3 sets of data.

We are ultimately interested in the response to the transporter knockouts and the absence of condition specific measurements, substrate uptake was refined to only account for metabolites that were mapped from Recon3D to the experimental data. Metabolomic data constraints test with flux variability analysis (FVA)^73^ to classify metabolites into three groups, those that can only be secreted, those that can only be taken up, and those that can be secreted or taken up. Interpretation of the metabolomic fold change measurements (KO relative to WT) were as follows:

- If KO/WT > 1 and the metabolite could only be secreted, then the metabolite exchange was constrained with a non-zero lower bound (10% of maximum secretion)
- If KO/WT > 1 and the metabolite could only be taken up, then the metabolite exchange was constrained with a lower bound that was greater than the minimum
- If KO/WT < 1 and the metabolite could only be secreted, then the metabolite exchange was constrained with an upper bound that was less than the maximum
- If KO/WT < 1 and the metabolite could only be taken up, then the metabolite exchange was constrained with upper bound that was less than zero (90% of maximum uptake).

Since the comparison is targeted at looking at alterations, an arbitrary reference uptake of 0.25 mM/gDW/hour was specified for the OAT WT models. The metabolomic constraints for the set of metabolites that were significantly altered in either OAT1 or OAT3 knockouts were applied as described above. Additionally ‘free’ uptake was permitted for oxygen, sodium, potassium, iron, magnesium, bicarbonate, protons, and water.

Cell specificity for metabolic genome reconstructions remains a challenge in terms of achieving high sensitivity and specificity^74^. An advantage of GIMME is that it does not assume that flux is correlated with levels of gene expression; this is generally more biologically appropriate in comparison to other approaches that may assume a quantitative (linear) correlation between transcription and enzymatic flux. A limitation of GIMME (also shared by other context-specific constraining methods), is that frequently non-tissue specific genes may be included when tissue profiling is performed across an entire organ (so it becomes an amalgam of tissues). We leveraged this potential weakness as a strength for the current study, in order to enable detection of potential interactions via the plasma metabolome with different organs (see sub-section “Tissue specific reactions (TSEA) and analysis”).

GIMME models were evaluated for variety of constraining objective functions, including biomass, urea, and ATP production. Model content did not vary significantly (fewer than 10 reactions) in reaction content when using the other objectives. ATP production was used, given that the transcriptomic data analysis was performed on the organ level (as opposed to single-cell) transcriptomics. Following construction of four GIMME models (WT and KO for OAT1 and OAT3) and merging with the microbiome model, MB, the feasible solution space of each model was sampled. In order to ensure microbiome viability, a small non-zero lower bound was applied for the microbial biomass vector.

Differentially active reactions between WT and KO were than computed from the normalized sampled feasible flux states^75^, requiring the following conditions: p < 0.001 for Kolmogorov–Smirnov test as well as one-way ANOVA F-test, with at least ≥ twofold change (or ≤ 0.5 fold change).

The gpSampler in the CobraToolbox was used to characterize the steady state solution space with 2n sampled points (for n reactions in the model). FBA can be used to define functional, context-specific subsystems, or “reaction co-sets”—groups of reactions with tightly correlated fluxes^76, 77^. These co-sets are of particular interest since they can be used to understand complex metabolic pathway dependencies, such as host-microbiome interactions. Reaction co-sets were calculated from these points with a 0.95 Pearson correlation coefficient cutoff. Model modifications and simulations were carried out with using CobraPy^78^ and the CobraToolbox v2.0^72^ with the Gurobi Optimizer (v 8.0). Network pathway maps were created using Escher Maps^79^.

### Tissue-specific reactions and analysis

Human metabolic reconstructions are built upon the known/established biochemical functions across all cells; thus, the construction of cell, tissue, and organ specific models can be performed through the specification of appropriate demands on metabolites. A recognized limitation of interpretation of genome-scale models has been the inability to assign causality in plasma metabolome sampling, due to the ability to interact with multiple organs. Since most tissues express the large majority of metabolic enzymes, we felt it was important to use a reconstruction independent methodology and dataset in order to define tissue specific subsets of genes for the purpose of subsequent genome-scale metabolic network analysis. In order to approximate the cellular metabolic objectives of different tissues, we used Tissue-Specific Enrichment Analysis (TSEA)^80^. Here we specifically refer to organs rather than tissues in order to highlight the more general nature of the metabolic objectives. For each model, TSEA was applied to the gene set and the genes corresponding to kidney, liver, colon, adipose tissue, skin, and brain were selected following Benjamini–Hochberg correction with p < 0.05. The corresponding reactions were then selected (using the *rxnGeneMat* matrix field in the *model* structure in Matlab) and the uniformly weighted linear sum of the reactions was used to define metabolic demand for each organ.

### Flux cones

3D phase planes for metabolic organ objectives of interest were constructed. For a triplet of reactions of interest, {v_i_, v_j_, v_k_} $\in$ v and i, j, k $\in {N}_{+}^{n}$, where v is the set of all network reaction vectors and n is the number of reactions in the network, and the set of all ordered permutations of {i,j,k}$\in$ P for is represented as P. Pseudo-code for the bi-level optimization problem follows,
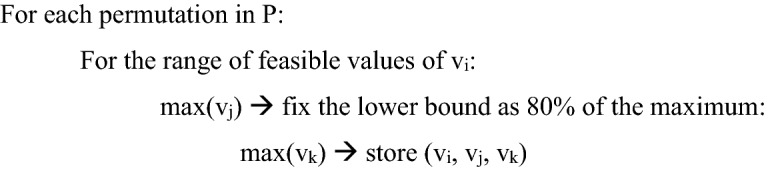


The resulting matrix (v_i_, v_j_, v_k_) was then plotted as a 3D flux phase plane.

### Circos plots

Circos plots^81^ were generated following calculation of pairwise interactions between competing organ metabolic objectives. The purpose was to identify functional metabolic dependencies under ‘normal’ conditions as well as OAT knockout to see how systemic metabolic states are affected by loss of OAT function. Comparisons were made following calculation of a matrix, M, whose entries m_ij_ are defined as max(c_j_) for i = j, and max(c_i_) for i $\ne$ j, with i, j $\in {N}_{+}^{n}$ , and v_j_^l^ = α*max(c_j_) for j subset of the reaction list. For each vector, a set is calculated covering a range of α ∈ (0,1]. Here, we used the standard definition for the objective vector, c (in contrast to GIMME formulation above), c ∈ {0,1}^n^ with $\sum_{i=1}^{n}{c}_{i}=1$.

Interpretation of Circos plots: Each plot should be compared with respect to a ‘reference’ organ that has a fixed lower bound (denoted single capital letter abbreviations: A: adipose, B: brain, C: colon, K: kidney, L: liver, S: skin, MB: microbiome). The lower bound minimum was set to 80% of the corresponding organ objective. Pairwise organ-organ optimizations were linear from 10 to 90%, so the selection of the lower bound is relatively independent of qualitative interpretations of results. For example, in the OAT1WT model for skin_lb, there are essentially equally sized ribbons extending to the different tissues (adipose, brain, colon, kidney, liver, skin) and microbiome. The *organ* to *organ*_lb ribbon can be considered the reference point. So, for example, if the ribbon width of organ1_lb to organ2 is the same as the width of the ribbon from organ1_lb to organ1, then it can be stated that organ1 and organ2 have essentially independent metabolic objectives. Conversely, if the width of the ribbon from organ1_lb to organ3 is very narrow (< 10%), then it can be concluded that they are metabolically competing objectives. Constrained organs (“_lb” post-fix) that have large ribbons to some tissues and small ribbons to others indicated organ-organ interactions that are independent of one another versus competitive with one another, respectively. Comparison of the WT and KO organ-dependency Circos plots identifies changes in the ‘normal’ pairwise tissue interactions that result as a consequent *Oat1* or *Oat3* knockout.

### Figures/illustrations

Circos plots were generated with Circos version 0.69–6 as cited above^81^. Data plots and phase planes were generated with Matlab R2016b (MathWorks, Natick, MA). All other figures were generated in Adobe Illustrator CC 2015 (Adobe Inc, San Jose, CA).

## Supplementary Information

Supplementary Figures.

Supplementary Tables.

## Data Availability

All data generated or analyzed during this study are included in this published article, its supplementary information files, and cited references.

## Abbreviations

ABC: ATP binding cassette
ADME: (Drug) Absorption, distribution, metabolism and elimination
ANOVA: Analysis of variance
BCRP: Breast cancer resistance protein
CKD: Chronic Kidney Disease
COBRA: Constraint-based reconstruction analysis
Da: Dalton
DMI: Drug-metabolite interactions
FBA: Flux balance analysis
FDA: Food and Drug Administration
FVA: Flux variability analysis
HIV: Human Immunodeficiency Virus
KO: Knockout
MOMR: Multi-organ metabolic reconstructions
MATE2: Multidrug and toxin extrusion protein 2
MRP2: Multidrug resistance related protein 2
NKT: Novel kidney transcript
NSAID: Non-steroidal anti-inflammatory drugs
OAT1: Organic anion transporter 1
OAT3: Organic anion transporter 3
OCTN2: Organic cation/carnitine transporter 2
QSP: Quantitative systems pharmacology
RSST: Remote sensing and signaling theory
SLC: Solute carrier family
TCA: Tricarboxylic acid
TSEA: Tissue specific expression analysis
WT: Wildtype
4CRSOLt_mb: P-cresol transport (microbiome)
ADD_mb: Adenine deaminase (microbiome)
ADE: Adenine
ADMDC_mb: Adenosylmethionine decarboxylase (microbiome)
AKG: Alpha ketoglutarate
ALATA_L: l-Alanine transaminase
ALKP_mb: Alkaline phosphatase (microbiome)
ATPS4r_mb: ATP synthase (microbiome)
DHA: Dihydroxyacetone
DHAP: Dihydroxyacetone phosphate
DHAPtr: Dihydroxyacetone phosphate transport
DHAt_mb: Dihydroxyacetone transport (microbiome)
FBA_mb: Fructose-bisphosphate aldolase (microbiome)
G3P: Glyceraldehyde 3-phosphate
GAPD_mb: Glyceraldehyde-3-phosphate dehydrogenase (microbiome)
GLU-L: L-glutamate
GLYALDDr: d-Glyceraldehyde dehydrogenase
HPYRRy: Hydroxypyruvate reductase (NADPH)
HXAND_mb: Hypoxanthine dehydrogenase (microbiome)
Met-L: l-methionine
MTAN_mb: Methylthioadenosine nucleosidase (microbiome)
PAPS: 3-Phosphoadenylyl sulfate
PCRESOLup: P-cresol uptake
PCS: P-cresol sulfate
PCSF: P-cresol sulfation
PFK_mb: Phosphofructokinase (microbiome)
PGCD: Phosphoglycerate dehydrogenase
PGK_mb: Phosphoglycerate kinase (microbiome)
PPM_mb: Phosphopentomutase (microbiome)
PPM: Phosphopentomutase
PSERT: Phosphoserine transaminase
PSP_L: Phosphoserine phosphatase (l-serine)
PYK_mb: Pyruvate kinase (microbiome)
PYR: Pyruvate
r0242: Glycerone phosphate phosphohydrolase
r0249: d-Ribose-5-phosphate ketol-isomerase
r0840: R5P ER to cytosol transport
r0841: RU5P ER to cytosol transport
R1P: Ribose-1-phosphate
RPE_mb: Ribulose 5-Phosphate 3-Epimerase (microbiome)
RPEc: Ribulose 5-Phosphate 3-Epimerase
RPI_mb: Ribose phosphate isomerase (microbiome)
SPMS_mb: Spermidine synthase (microbiome)
TALA_mb: Transaldolase (microbiome)
TKT1_mb: Transketolase (microbiome)
TKT1: Transketolase
TKT2_mb: Transketolase (microbiome)
TKT2: Transketolase
TPI_mb: Triose-phosphate isomerase (microbiome)
TPI: Triose-phosphate isomerase
TRIOK: Triokinase
TYMSULT: Tyramine sulfotransferase,
Tyr-L: l-tyrosine
TYRL_mb: Tyrosine lyase (microbiome)
XAN: Xanthine
XANt: Xanthine reversible transport
XANt2_mb: Xanthine transport (microbiome)

## References

[1] Alicic RZ, Johnson EJ, Tuttle KR (2018). SGLT2 inhibition for the prevention and treatment of diabetic kidney disease: A review. Am. J. Kidney Dis..

[2] Ivanyuk A, Livio F, Biollaz J, Buclin T (2017). Renal drug transporters and drug interactions. Clin. Pharmacokinet..

[3] Nigam SK (2018). The SLC22 Transporter family: A paradigm for the impact of drug transporters on metabolic pathways, signaling, and disease. Annu. Rev. Pharmacol. Toxicol..

[4] Lopez-Nieto CE, You G, Bush KT, Barros EJ, Beier DR, Nigam SK (1997). Molecular cloning and characterization of NKT, a gene product related to the organic cation transporter family that is almost exclusively expressed in the kidney. J. Biol. Chem..

[5] Nigam SK (2015). What do drug transporters really do?. Nat. Rev. Drug Discov..

[6] Ishikawa T (2009). Emerging trends in human ABC transporters. Pharm. Res..

[7] Bush KT, Singh P, Nigam SK (2020). Gut-derived uremic toxin handling in vivo requires OAT-mediated tubular secretion in chronic kidney disease. JCI Insight.

[8] Nigam SK, Bhatnagar V (2018). The systems biology of uric acid transporters: The role of remote sensing and signaling. Curr. Opin. Nephrol. Hypertens..

[9] Ingraham L, Li M, Renfro JL, Parker S, Vapurcuyan A, Hanna I (2014). A plasma concentration of alpha-ketoglutarate influences the kinetic interaction of ligands with organic anion transporter 1. Mol. Pharmacol..

[10] Nigam SK, Bush KT, Bhatnagar V, Poloyac SM, Momper JD (2020). The systems biology of drug metabolizing enzymes and transporters: Relevance to quantitative systems pharmacology. Clin. Pharmacol. Ther..

[11] Dong F, Perdew GH (2020). The aryl hydrocarbon receptor as a mediator of host-microbiota interplay. Gut Microbes..

[12] Jansen J, Jansen K, Neven E, Poesen R, Othman A, van Mil A (2019). Remote sensing and signaling in kidney proximal tubules stimulates gut microbiome-derived organic anion secretion. Proc. Natl. Acad. Sci. U.S.A..

[13] Mo ML, Jamshidi N, Palsson BO (2007). A genome-scale, constraint-based approach to systems biology of human metabolism. Mol. Biosyst..

[14] Reed JL, Famili I, Thiele I, Palsson BO (2006). Towards multidimensional genome annotation. Nat. Rev. Genet..

[15] Becker SA, Palsson BO (2008). Context-specific metabolic networks are consistent with experiments. PLoS Comput. Biol..

[16] Lewis NE, Nagarajan H, Palsson BO (2012). Constraining the metabolic genotype-phenotype relationship using a phylogeny of in silico methods. Nat. Rev. Microbiol..

[17] Frezza C, Zheng L, Folger O, Rajagopalan KN, MacKenzie ED, Jerby L (2011). Haem oxygenase is synthetically lethal with the tumour suppressor fumarate hydratase. Nature.

[18] Gatto F, Nookaew I, Nielsen J (2014). Chromosome 3p loss of heterozygosity is associated with a unique metabolic network in clear cell renal carcinoma. Proc. Natl. Acad. Sci. U.S.A..

[19] Jerby L, Shlomi T, Ruppin E (2010). Computational reconstruction of tissue-specific metabolic models: Application to human liver metabolism. Mol. Syst. Biol..

[20] Mardinoglu A, Agren R, Kampf C, Asplund A, Uhlen M, Nielsen J (2014). Genome-scale metabolic modelling of hepatocytes reveals serine deficiency in patients with non-alcoholic fatty liver disease. Nat. Commun..

[21] Thomas A, Rahmanian S, Bordbar A, Palsson BO, Jamshidi N (2014). Network reconstruction of platelet metabolism identifies metabolic signature for aspirin resistance. Sci. Rep..

[22] Ahn SY, Jamshidi N, Mo ML, Wu W, Eraly SA, Dnyanmote A (2011). Linkage of organic anion transporter-1 to metabolic pathways through integrated "omics"-driven network and functional analysis. J. Biol. Chem..

[23] Liu HC, Jamshidi N, Chen Y, Eraly SA, Cho SY, Bhatnagar V (2016). An Organic Anion Transporter 1 (OAT1)-centered metabolic network. J. Biol. Chem..

[24] Wu W, Jamshidi N, Eraly SA, Liu HC, Bush KT, Palsson BO (2013). Multispecific drug transporter Slc22a8 (Oat3) regulates multiple metabolic and signaling pathways. Drug Metab. Dispos..

[25] Eraly SA, Liu HC, Jamshidi N, Nigam SK (2015). Transcriptome-based reconstructions from the murine knockout suggest involvement of the urate transporter, URAT1 (slc22a12), in novel metabolic pathways. Biochem. Biophys. Rep..

[26] Brunk E, Sahoo S, Zielinski DC, Altunkaya A, Drager A, Mih N (2018). Recon3D enables a three-dimensional view of gene variation in human metabolism. Nat. Biotechnol..

[27] Eraly SA, Monte JC, Nigam SK (2004). Novel slc22 transporter homologs in fly, worm, and human clarify the phylogeny of organic anion and cation transporters. Physiol. Genom..

[28] Vallon V, Rieg T, Ahn SY, Wu W, Eraly SA, Nigam SK (2008). Overlapping in vitro and in vivo specificities of the organic anion transporters OAT1 and OAT3 for loop and thiazide diuretics. Am. J. Physiol. Renal Physiol..

[29] Bush KT, Wu W, Lun C, Nigam SK (2017). The drug transporter OAT3 (SLC22A8) and endogenous metabolite communication via the gut-liver-kidney axis. J. Biol. Chem..

[30] Nigam AK, Li JG, Lall K, Shi D, Bush KT, Bhatnagar V (2020). Unique metabolite preferences of the drug transporters OAT1 and OAT3 analyzed by machine learning. J. Biol. Chem..

[31] Vriend J, Hoogstraten CA, Venrooij KR, van den Berge BT, Govers LP, van Rooij A (2019). Organic anion transporters 1 and 3 influence cellular energy metabolism in renal proximal tubule cells. Biol. Chem..

[32] Nigam SK, Bush KT, Martovetsky G, Ahn SY, Liu HC, Richard E (2015). The organic anion transporter (OAT) family: A systems biology perspective. Physiol Rev..

[33] Engelhart DC, Azad P, Ali S, Granados JC, Haddad GG, Nigam SK (2020). Drosophila SLC22 orthologs related to OATs, OCTs, and OCTNs regulate development and responsiveness to oxidative stress. Int. J. Mol. Sci..

[34] Sweet DH, Chan LM, Walden R, Yang XP, Miller DS, Pritchard JB (2003). Organic anion transporter 3 (Slc22a8) is a dicarboxylate exchanger indirectly coupled to the Na+ gradient. Am. J. Physiol. Renal Physiol..

[35] Mangelsdorf DJ, Thummel C, Beato M, Herrlich P, Schutz G, Umesono K (1995). The nuclear receptor superfamily: The second decade. Cell.

[36] Rosenthal SB, Bush KT, Nigam SK (2019). A network of SLC and ABC transporter and DME genes involved in remote sensing and signaling in the gut-liver-kidney axis. Sci. Rep..

[37] Lowenstein J, Nigam SK (2021). Uremic toxins in organ crosstalk. Front. Med. (Lausanne)..

[38] Nigam SK, Bush KT (2019). Uraemic syndrome of chronic kidney disease: Altered remote sensing and signalling. Nat. Rev. Nephrol..

[39] Gallegos TF, Martovetsky G, Kouznetsova V, Bush KT, Nigam SK (2012). Organic anion and cation SLC22 "drug" transporter (Oat1, Oat3, and Oct1) regulation during development and maturation of the kidney proximal tubule. PLoS ONE.

[40] Sweeney DE, Vallon V, Rieg T, Wu W, Gallegos TF, Nigam SK (2011). Functional maturation of drug transporters in the developing, neonatal, and postnatal kidney. Mol. Pharmacol..

[41] Dougherty JD, Schmidt EF, Nakajima M, Heintz N (2010). Analytical approaches to RNA profiling data for the identification of genes enriched in specific cells. Nucleic Acids Res..

[42] Ademola JI, Wester RC, Maibach HI (1993). Metabolism of 3-indolylacetic acid during percutaneous absorption in human skin. J. Pharm. Sci..

[43] Svensson CK (2009). Biotransformation of drugs in human skin. Drug Metabol. Dispos..

[44] Tobin DJ (2006). Biochemistry of human skin–our brain on the outside. Chem. Soc. Rev..

[45] Momper JD, Tsunoda SM, Ma JD (2016). Evaluation of proposed in vivo probe substrates and inhibitors for phenotyping transporter activity in humans. J. Clin. Pharmacol..

[46] Murray M, Zhou F (2017). Trafficking and other regulatory mechanisms for organic anion transporting polypeptides and organic anion transporters that modulate cellular drug and xenobiotic influx and that are dysregulated in disease. Br. J. Pharmacol..

[47] Emami Riedmaier A, Burk O, van Eijck BA, Schaeffeler E, Klein K, Fehr S (2016). Variability in hepatic expression of organic anion transporter 7/SLC22A9, a novel pravastatin uptake transporter: Impact of genetic and regulatory factors. Pharmacogenom. J..

[48] Zhu C, Nigam KB, Date RC, Bush KT, Springer SA, Saier MH (2015). Evolutionary analysis and classification of OATs, OCTs, OCTNs, and other SLC22 transporters: Structure-function implications and analysis of sequence motifs. PLoS ONE.

[49] Aslamkhan AG, Thompson DM, Perry JL, Bleasby K, Wolff NA, Barros S (2006). The flounder organic anion transporter fOat has sequence, function, and substrate specificity similarity to both mammalian Oat1 and Oat3. Am. J. Physiol. Regul. Integr. Comp. Physiol..

[50] Engelhart DC, Granados JC, Shi D, Saier MH (2020). Systems biology analysis reveals eight SLC22 transporter subgroups, including OATs, OCTs, and OCTNs. Int. J. Mol. Sci..

[51] Granados JC, Bhatnagar V, Nigam SK (2022). Blockade of organic anion transport in humans after treatment with the drug probenecid leads to major metabolic alterations in plasma and urine. Clin. Pharmacol. Ther..

[52] Bleasby K, Hall LA, Perry JL, Mohrenweiser HW, Pritchard JB (2005). Functional consequences of single nucleotide polymorphisms in the human organic anion transporter hOAT1 (SLC22A6). J. Pharmacol. Exp. Ther..

[53] Wu W, Bush KT, Nigam SK (2017). Key role for the organic anion transporters, OAT1 and OAT3, in the in vivo handling of uremic toxins and solutes. Sci. Rep..

[54] Deguchi T, Takemoto M, Uehara N, Lindup WE, Suenaga A, Otagiri M (2005). Renal clearance of endogenous hippurate correlates with expression levels of renal organic anion transporters in uremic rats. J. Pharmacol. Exp. Ther..

[55] Miyamoto Y, Watanabe H, Noguchi T, Kotani S, Nakajima M, Kadowaki D (2011). Organic anion transporters play an important role in the uptake of p-cresyl sulfate, a uremic toxin, in the kidney. Nephrol. Dial.. Transplant..

[56] Wikoff WR, Nagle MA, Kouznetsova VL, Tsigelny IF, Nigam SK (2011). Untargeted metabolomics identifies enterobiome metabolites and putative uremic toxins as substrates of organic anion transporter 1 (Oat1). J. Proteome Res..

[57] Nigam SK, Wu W, Bush KT, Hoenig MP, Blantz RC, Bhatnagar V (2015). Handling of drugs, metabolites, and uremic toxins by kidney proximal tubule drug transporters. Clin. J. Am. Soc. Nephrol..

[58] Nielsen MMK, Aryal E, Safari E, Mojsoska B, Jenssen H, Prabhala BK (2021). Current state of SLC and ABC transporters in the skin and their relation to sweat metabolites and skin diseases. Proteomes.

[59] Jamshidi N, Xu X, von Lohneysen K, Soldau K, Mohney RP, Karoly ED (2020). Metabolome changes during in vivo red cell aging reveal disruption of key metabolic pathways. iScience.

[60] Nigam SK, Granados JC (2022). A biological basis for pharmacokinetics: The remote sensing and signaling theory. Clin. Pharmacol. Ther..

[61] Gleeson MP, Hersey A, Hannongbua S (2011). In-silico ADME models: A general assessment of their utility in drug discovery applications. Curr. Top. Med. Chem..

[62] Knight-Schrijver VR, Chelliah V, Cucurull-Sanchez L, Le Novere N (2016). The promises of quantitative systems pharmacology modelling for drug development. Comput. Struct. Biotechnol. J..

[63] Zhang P, Azad P, Engelhart DC, Haddad GG, Nigam SK (2021). SLC22 transporters in the fly renal system regulate response to oxidative stress in vivo. Int. J. Mol. Sci..

[64] Granados JC, Nigam AK, Bush KT, Jamshidi N, Nigam SK (2021). A key role for the transporter OAT1 in systemic lipid metabolism. J. Biol. Chem..

[65] Granados JC, Richelle A, Gutierrez JM, Zhang P, Zhang X, Bhatnagar V (2021). Coordinate regulation of systemic and kidney tryptophan metabolism by the drug transporters OAT1 and OAT3. J. Biol. Chem..

[66] Bhatnagar V, Richard EL, Wu W, Nievergelt CM, Lipkowitz MS, Jeff J (2016). Analysis of ABCG2 and other urate transporters in uric acid homeostasis in chronic kidney disease: Potential role of remote sensing and signaling. Clin. Kidney J..

[67] Oh YK, Palsson BO, Park SM, Schilling CH, Mahadevan R (2007). Genome-scale reconstruction of metabolic network in *Bacillus subtilis* based on high-throughput phenotyping and gene essentiality data. J. Biol. Chem..

[68] Flahaut NA, Wiersma A, van de Bunt B, Martens DE, Schaap PJ, Sijtsma L (2013). Genome-scale metabolic model for Lactococcus lactis MG1363 and its application to the analysis of flavor formation. Appl. Microbiol. Biotechnol..

[69] Feist AM, Henry CS, Reed JL, Krummenacker M, Joyce AR, Karp PD (2007). A genome-scale metabolic reconstruction for *Escherichia coli* K-12 MG1655 that accounts for 1260 ORFs and thermodynamic information. Mol. Syst. Biol..

[70] Monk JM, Charusanti P, Aziz RK, Lerman JA, Premyodhin N, Orth JD (2013). Genome-scale metabolic reconstructions of multiple *Escherichia coli* strains highlight strain-specific adaptations to nutritional environments. Proc. Natl. Acad. Sci. U.S.A..

[71] Monk JM, Lloyd CJ, Brunk E, Mih N, Sastry A, King Z (2017). iML1515, a knowledgebase that computes *Escherichia coli* traits. Nat. Biotechnol..

[72] Schellenberger J, Que R, Fleming RM, Thiele I, Orth JD, Feist AM (2011). Quantitative prediction of cellular metabolism with constraint-based models: The COBRA Toolbox v2.0. Nat. Protoc..

[73] Mahadevan R, Schilling CH (2003). The effects of alternate optimal solutions in constraint-based genome-scale metabolic models. Metab. Eng..

[74] Opdam S, Richelle A, Kellman B, Li S, Zielinski DC, Lewis NE (2017). A systematic evaluation of methods for tailoring genome-scale metabolic models. Cell Syst..

[75] Bordbar A, Lewis NE, Schellenberger J, Palsson BO, Jamshidi N (2010). Insight into human alveolar macrophage and *M. tuberculosis* interactions via metabolic reconstructions. Mol. Syst. Biol..

[76] Jamshidi N, Palsson BO (2006). Systems biology of SNPs. Mol. Syst. Biol..

[77] Papin JA, Reed JL, Palsson BO (2004). Hierarchical thinking in network biology: The unbiased modularization of biochemical networks. Trends Biochem. Sci..

[78] Ebrahim A, Lerman JA, Palsson BO, Hyduke DR (2013). COBRApy: COnstraints-based reconstruction and analysis for python. BMC Syst. Biol..

[79] King ZA, Drager A, Ebrahim A, Sonnenschein N, Lewis NE, Palsson BO (2015). Escher: A web application for building, sharing, and embedding data-rich visualizations of biological pathways. PLoS Comput. Biol..

[80] Xu X, Wells AB, O'Brien DR, Nehorai A, Dougherty JD (2014). Cell type-specific expression analysis to identify putative cellular mechanisms for neurogenetic disorders. J. Neurosci..

[81] Krzywinski M, Schein J, Birol I, Connors J, Gascoyne R, Horsman D (2009). Circos: An information aesthetic for comparative genomics. Genome Res..

