# Supplementary material for: Drug transporters OAT1 and OAT3 have specific effects on multiple organs and gut microbiome as revealed by contextualized metabolic network reconstructions
Source: Sci Rep. 2022 Oct 31;12:18308. doi: 10.1038/s41598-022-21091-w (PMC9622871; doi:10.1038/s41598-022-21091-w)

Supplemental Figures and Legends

Supplemental Figure 1

Schematic of the data analysis workflow. The NCBI Homologene Database was used to map human transcripts to mouse (generating Recon3Dmm from Recon3D). The subsequent steps in data integration and analysis correspond to Figures 1 and 2 in the main text.

Supplemental Figure 2

Content comparison between WT and KO models for OAT1 and OAT3. The pie charts provide a comparison of the relative size of the OAT1 and OAT3 WT and KO intracellular and microbiome compartments according to the number of metabolites.

Supplemental Figure 3

Organ interaction plots for OAT1 WT and KO models. (A) Wildtype circos plot of organ-organ interactions with ribbon width corresponding to the maximum percentage of an organ's metabolic objective while maintaining at least 80% of the maximum for another organ. (B) Knockout Circos plot of organ-organ interactions. (C) Different (in percentages) between the WT and KO Circos plots.

Supplemental Figure 1

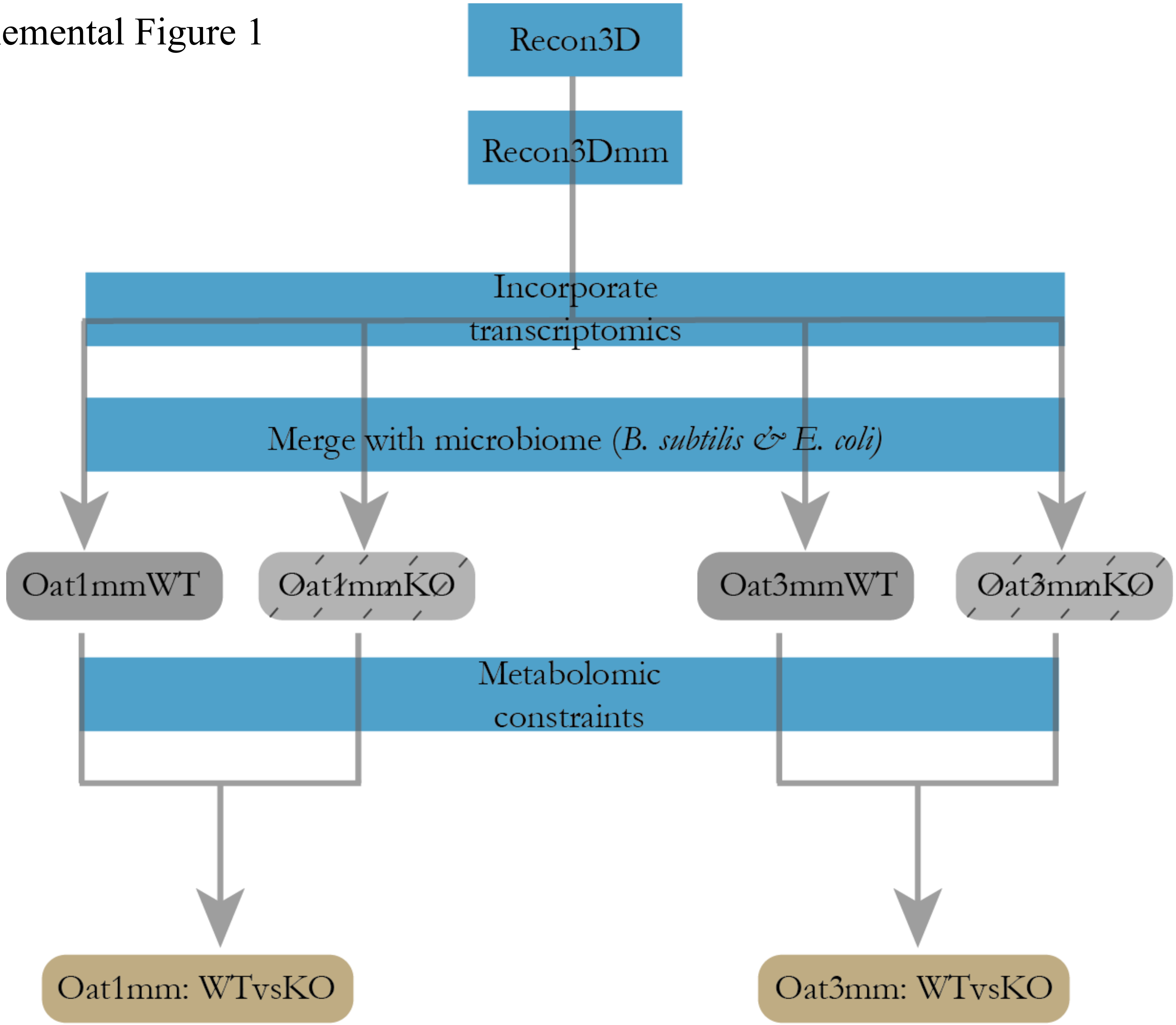

Supplemental Figure 2

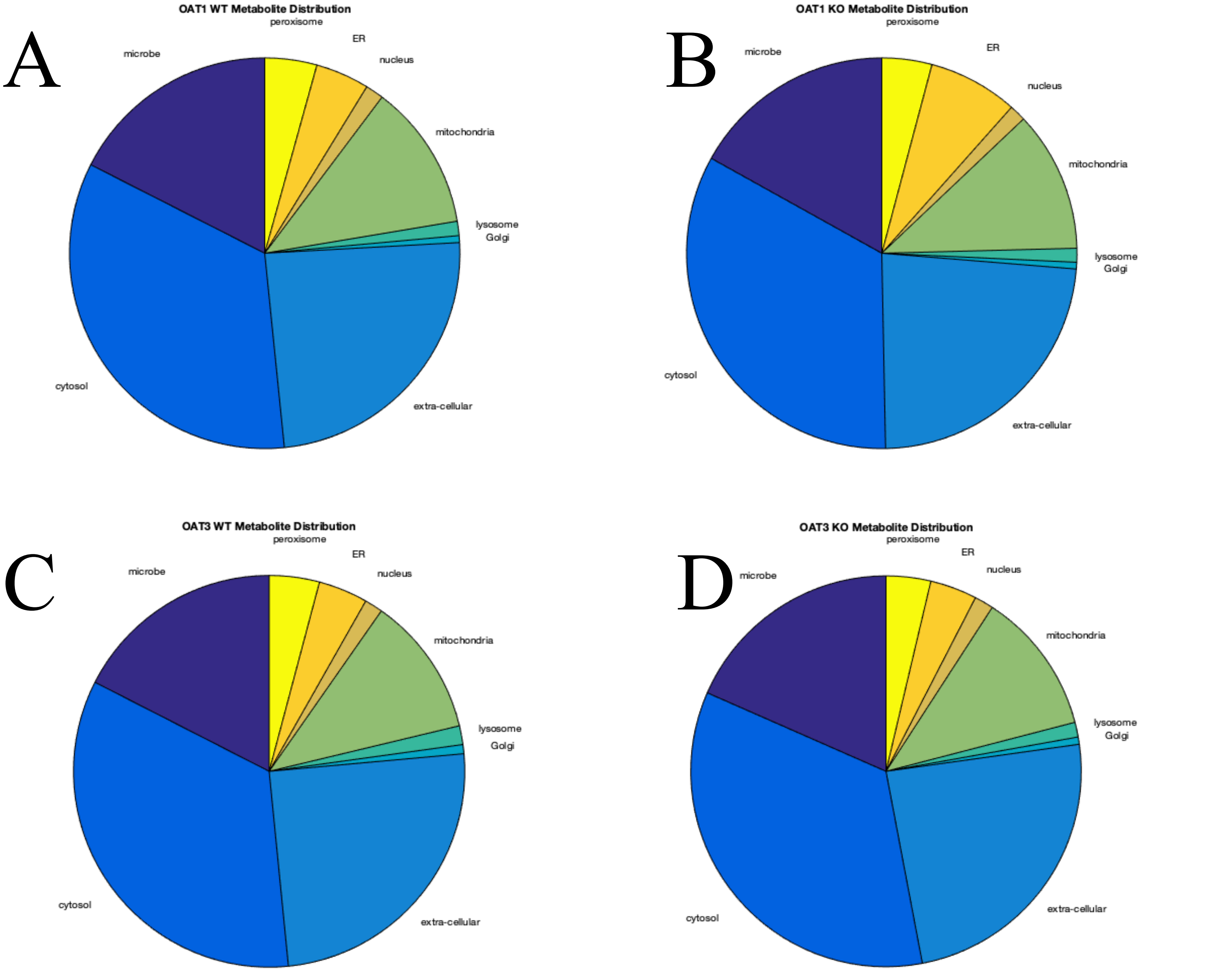

Supplemental Figure 3

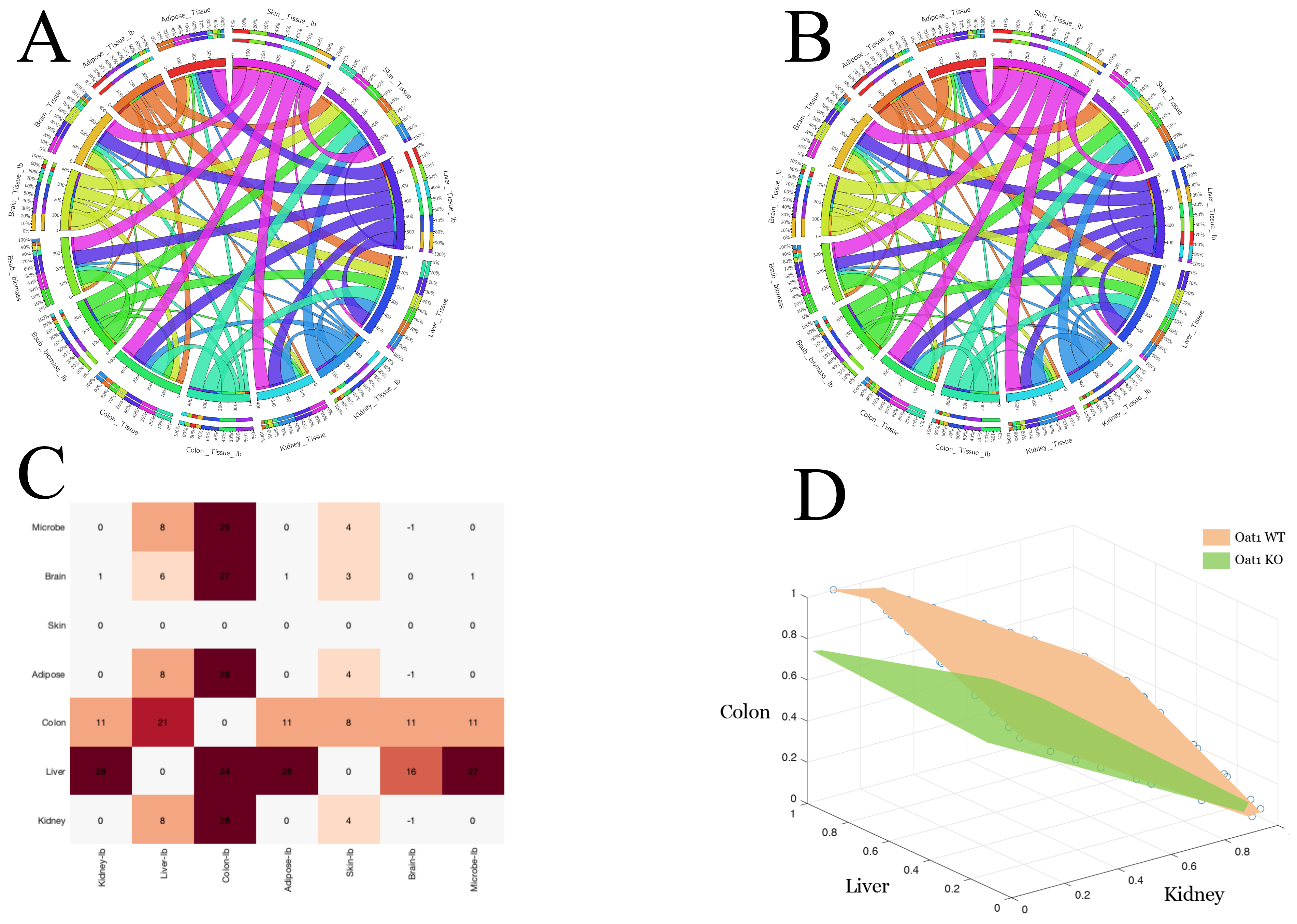

Supplement: Supplementary file 1 — Supplementary Figures. [file 41598_2022_21091_MOESM1_ESM.pdf]
